# *Globisporangium tabrizense* sp. nov., *Globisporangium mahabadense* sp. nov., and *Pythium bostanabadense* sp. nov. (Oomycota), three new species from Iranian aquatic environments

**DOI:** 10.1038/s41598-024-81651-0

**Published:** 2024-12-30

**Authors:** Reza Ahadi, Ali Chenari Bouket, Alireza Alizadeh, Hossein Masigol, Hans-Peter Grossart

**Affiliations:** 1 Department of Plant Protection, Faculty of Agriculture, Azarbaijan Shahid Madani University, Tabriz, 5375171379 Iran; 2 Plant Protection Research Department, East Azarbaijan Agricultural and Natural Resources Research and Education Centre, Agricultural Research, Education and Extension Organization (AREEO), Tabriz, 5355179854 Iran; 3 Plankton and Microbial Ecology, Leibniz Institute for Freshwater Ecology and Inland Fisheries (IGB), Berlin, Germany; 4 Institute of Biochemistry and Biology, University of Potsdam, Potsdam, Germany

**Keywords:** Biodiversity, Oomycetes, Pathogens, Phylogeny, *Pythiaceae*, Water molds, Biodiversity, Freshwater ecology

## Abstract

During a survey on the biodiversity of oomycetes in aquatic environments in northwest Iran (East Azarbaijan and West Azarbaijan provinces), three *Pythium* and four *Globisporangium* isolates were recovered from agricultural water pools and irrigation canals, respectively. Through a polyphasic approach combining morphology and phylogenetic analysis using the nuclear rDNA ITS1-5.8 S-ITS2 (ITS) and partial sequences of the cytochrome *c* oxidase subunit I and II (*COX1* and *COX2*), three novel species were identified namely *Globisporangium tabrizense* sp. nov., *G. mahabadense* sp. nov., and *Pythium bostanabadense* sp. nov. Furthermore, experiments confirmed the pathogenicity of all identified species on cucumber seedlings, suggesting a pathogenic lifestyle also in aquatic systems. Our research contributes to a better understanding of the diversity, host range and distribution of oomycetes genera *Globisporangium* and *Pythium* in northwestern Iran. Detailed morphological descriptions and illustrations are provided for all species.

**Supplementary Information:**

The online version contains supplementary material available at 10.1038/s41598-024-81651-0.

## Introduction

Oomycetes are a diverse group within the kingdom Straminipila and infamous for their devastating pathogenic capacity against agricultural plants^1^. *Phytophthora infestans* (Mont.) de Bary (causal agent of late blight of potato), *Plasmopara viticola* (Berk. & M.A. Curtis) Berl. & De Toni (downy mildew of grape), and *Albugo candida* (Pers. ex J.F. Gmel.) Roussel (white rust of crucifers) are three major oomycete plant pathogens responsible for major economic losses and serious damage to agricultural ecosystems^2^. However, oomycetes also dominate various aquatic environments worldwide, ranging from freshwater to marine ecosystems^3^. Characterized by filamentous hyphal growth, they fulfill crucial ecological roles, serving as decomposers^4^ and/or parasites of aquatic animals, thereby influencing both biotic and abiotic components of aquatic habitats. As for decomposers, Masigol et al.^5–7^ showed that members of the order *Saprolegniales* such as *Achlya* Nees and *Dictyuchus* Leitg. are involved in the degradation of cellulose- and hemicellulose-like compounds in lagoon environments. Additionally, oomycetes comprise two notorious pathogens in freshwater ecosystems: *Aphanomyces astaci* Schikora and *Saprolegnia parasitica* Coker, responsible for the crayfish plague in crayfish^8^ and saprolegniosis in fish and amphibians^9^.

Despite their potential importance – also in aquatic ecosystems, the taxonomy and ecology of many aquatic oomycetes remain understudied, with numerous species likely yet to be discovered and described. *Pythium* Nees is a good example of such shortcomings as it has been studied biasedly due to its significance in plant pathology, particularly as a causal agent of damping-off disease in various crops^10^. As a result, its ecological roles and diversity in aquatic ecosystems have received relatively scant attention. However, more recently, several studies have reported various *Pythium* taxa in aquatic environments using both culture-dependent^11,12^ and culture-independent methods^13^ - though to a low extent, pointing out their largely untapped diversity and probably unknown functionality. Given its already known pathogenicity toward a broad range of plant hosts, one might hypothesize that *Pythium* acts as parasite of aquatic plants. For example, *Pythium phragmitis* is associated with the decline of the aquatic plant *Phragmites australis*^14,15^. Additionally, there are some speculations about aquatic *Pythium* spp. as parasites of copepods (e.g., *Daphnia pulex* and *Parabroteas sarsi* )^16,17^ and rotifers (e.g., *Asplanchna girodi*)^18^. Such pathogenic interactions are a matter of great ecological importance as copepods and rotifers are both primary consumers and food sources and facilitate energy transfer from primary producers to higher trophic levels^19^. Therefore, the first step toward a better understanding of what *Pythium* species might do in freshwater ecosystems is to investigate their compositional diversity and study their associations with various hosts.

Taxonomically speaking, *Pythium* belongs to the family *Pythiaceae*, the order *Pythiales*, and phylum *Oomycota*^20^. It can be well recognized via its various sporangia shapes and the development of zoospores within a vesicle at the discharge tube tip stemming from a sporangium^21^. Molecular phylogenetics revealed the paraphyletic nature of *Pythium* and proposed the genus to be split into several lineages^22^. *Pythium* was divided into 11 clades (A to K) by Lévesque and de Cock^23^ based on the nuclear rDNA internal transcribed spacer region ITS1–5.8 S–ITS2 (ITS barcode) and D1–D3 domains of nuclear 28 S rDNA phylogenies. Additionally, multigene phylogenetic approaches confirmed the separation of the *Pythium* clades into 10 clades^24^ with the placement of clade K in a newly created genus called *Phytopythium*^25^. Later, Uzuhashi et al.^26^ divided *Pythium sensu lato* into five genera namely *Pythium sensu stricto* (clades A-D), *Globisporangium* (clades E-G, I, and J), *Elongisporangium* (clade H), *Ovatisporangium* (clade K, *Phytopythium*), and *Pilasporangium* (distinct from the 11-lettered clades). Accuracy and constant improvement of the overall picture of *Pythium s.l.* diversity relies on more intense samplings from ecologically and geographically diverse locations as it has been the case for *Phytophthora* and its close relatives^27^. In fact, by exploring yet under-surveyed regions of the world, freshwater ecosystems in particular, we might be able to further reorganize the genus *Pythium s.l.*, resolve its polyphyletic nature more effectively, and finally unveil its true diversity.

Despite our knowledge of terrestrial pathogenic oomycetes in Iran, the distribution and ecology of aquatic taxa have been rarely investigated. With only a few studies on the distribution of saprophytic members of *Saprolegniales*^5,28^ in Iran and their functions in nutrient cycling^6,29–31^, little information is available on how diverse aquatic *Pythium s.l.* are and what functions they might have. Therefore, as part of an ongoing study of oomycetes species diversity, our manuscript aims to increase our limited knowledge of aquatic *Globisporangium* and *Pythium* in Iranian aquatic ecosystems. Three new species, *Globisporangium tabrizense* sp. nov., *G. mahabadense* sp. nov., and *Pythium bostanabadense* sp. nov., were isolated and described in terms of their phylogenetic placement using the ITS, *cox1*, and *cox2* regions, as well as their morphometric characteristics and pathogenic capabilities.

## Materials and methods

### Sample collection and isolation

Sampling was conducted in various aquatic environments across multiple locations within East and West Azerbaijan provinces, Iran. Samples included algae from agricultural water pools and irrigation canals, as well as roots of the grass species *Cynodon dactylon* growing within the agricultural irrigation canals. Samples were collected in 50 mL Falcon tubes and stored at 4 °C prior to processing in the plant pathology laboratory. Following surface sterilization with sterile distilled water, tissue samples were cultured on NARF^32^ (nystatin + ampicillin + rifampicin + fluazinam) agar, a semi-selective medium for oomycetes, and incubated at 15 °C for five days. Upon hyphal observation, a portion of the culture was transferred to WA medium (Agar 20.0 g/L) for isolate purification using the hyphal tip method^33^. Purified isolates were preserved on Corn Meal Agar in McCarthy vials at 10 °C.

## Morphological analysis

Colony characteristics and growth patterns of the isolates were observed two weeks after inoculation on various agar media including Corn Meal Agar^34^ (MIRMEDIA, Iran), Potato Dextrose Agar (PDA) (Sigma Aldrich, Germany), Malt Extract Agar (MEA) (DIFCO, USA), Potato Carrot Agar (PCA) (potatoes 20.0 g., carrots 20.0 g., and Agar 15.0 g/L (Sigma Aldrich, Germany)), and V8-juice Agar^35^ (SIGMA, Germany) at 25 °C. Morphological evaluations were performed on sexual and asexual structures produced on autoclaved hempseeds and ryegrass pieces floating in sterile water from different sources (pond water, distilled water, tap water)^36^. Twenty measurements were taken for each structure observed. Microscopic structures were photographed using a Nikon-Eclipse Ti2 microscope with a digital camera system (Nikon, Japan). All purified cultures were deposited in the Fungal Culture Collection of Azarbaijan Shahid Madani University, Tabriz, Iran (AZFC) as well as the Iranian Fungal Culture Collection (IRAN) at the Iranian Research Institute of Plant Protection in Tehran. Type specimens were also deposited in the herbarium of the Iranian Research Institute of Plant Protection at the Iranian Research Institute of Plant Protection in Tehran, Iran.

The critical temperatures for growth were determined by incubating the strains on Potato Carrot Agar (PCA) at temperatures ranging 0, 2, 5, 10, 15, 20, 25, 30, 35, 40 °C, with three replicates. Descriptions were provided based on ex-type strains; additional data for strains showing distinct morphological differences was included.

## Phylogenetic analysis

Genomic DNA was extracted from five-days-grown oomycetes on CMA using a modified^37^ manual procedure. The 5.8 S nuclear ribosomal RNA gene, along with its two flanking internal transcribed spacers (ITS), and partial sequences of the *cox1* and *cox2* genes were amplified using the following primers: ITS5 (GGAAGTAAAAGTCGTAACAAGG) and ITS4 (TCCTCCGCTTATTGATATGC)^38^ for the ITS region; FM55 (GGCATACCAGCTAAACCTAA) and FM52R (TTAGAATGGAATTAGCACAAC)^39^ for the *cox1* region; and FM58 (CCACAAATTTCACTACATTGA) and FM66 (TAGGATTTCAAGATCCTGC)^39^ for the *cox2* region. All reactions were conducted in a total volume of 50 µL, containing 25 µL ready to use PCR Master mix MM2062 (SinaClon, Tehran, Iran), 1.2 µM of each primer, 18.6 µL DNase free Water and 10 ng DNA. Amplifications were carried out using a PeqStar 96X universal thermal cycler with the following condition for ITS-rDNA as 95 °C for five min followed by 30 cycles including denaturation at 95 °C for 30 s, annealing at 55 °C for 30 s and extension at 72 °C for one min, and a final extension step at 72 °C for seven min, and for *Cox1* and *Cox2* genes as 94 °C for five min followed by 40 cycles including denaturation at 94 °C for 30 s, annealing at 54 °C for 30 s and extension at 72 °C for one min, and a final extension step at 72 °C for seven min.

PCR amplicons were sequenced by Macrogen (Amsterdam, the Netherlands) using the amplifying primers. Raw sequences were manually assessed using SeqMan II^®^ (DNA STAR) and MEGA v. 6^40^. The sequences of the examined isolates for each genomic region were compared with other oomycetes DNA sequences using the blast tools of the NCBI GenBank database (www.ncbi.nlm.nih.gov/genbank/). Sequence data from ex-type and reference strains of known *Globisporangium* and *Pythium* species were obtained from NCBI GenBank (Supplementary Table S1). The retrieved sequences were assembled using Geneious (version 5.6) and aligned using the Q-INS-I algorithm in MAFFT in the latest version, available on the MAFFT web server^41,42^, separately for each of the genomic regions. Subsequently, after the removal of leading and trailing gaps, phylogenetic analyses were done on the TrEase webserver^43^ for the individual genes using FastTree2^44^ for Minimum Evolution, RAxML^45^ for Maximum Likelihood, and MrBayes^46^ for Bayesian inference, each in the latest version available. For the Bayesian analysis, a GTR model was selected and the analyses were run on random trees for 1,000,000 generations, discarding 30% of the first trees as burn-in steps of the analysis to determine posterior probabilities from the remaining trees. RAxML and FastTree2 trees were drawn by choosing GTRGAMMA and GTR algorithms, respectively, and the reliability of the inferred tree was estimated by bootstrap analysis with 1000 replications. After ensuring that there are no supported conflicting topologies in the phylogeny of the individual loci, they were concatenated, with the borders marked to ensure independent modeling of substitution rates for each partition. Multigene phylogenies (ITS, *cox1* and *cox2*) with support values were calculated in the same way mentioned above using three different approaches to assess the robustness of the inferred phylogenies. The sequences obtained in this study were deposited in GenBank and their accession numbers are given in Supplementary Table S1.

## Pathogenicity experiments

To assess the pathogenicity of the newly described *Globisporangium tabrizense* sp. nov., *G. mahabadense* sp. nov., and *Pythium bostanabadense* sp. nov., pathogenicity assays were conducted using a single isolate of each species (IRAN 4985 C, IRAN 4986 C, and IRAN 4989 C, respectively). Cucumber (*Cucumis sativus* L.), a known host for a wide range of oomycetes^21,47–50^, was selected as the test plant. Inoculum was prepared following the methods of Broders et al.^51^ and Chenari Bouket et al.^52^ with minor modifications. A sterile substrate containing sandy loam soil, wheat seed, and distilled water was inoculated with five mycelial plugs (5 mm³) from three–day–old PDA cultures of the target isolates. After nine days of incubation at 25 °C to allow for substrate colonization, cucumber seeds were sown and grown in a greenhouse at 25 ± 2 °C with a 16–hour photoperiod for 14 days.

Positive controls consisted of plants grown in soil inoculated with each of the test isolates, while negative controls were grown in non-inoculated soil. Symptoms including wilting, crown and root rot, and stem discoloration and deterioration were assessed daily for 14 days. Symptomatic plants were further analyzed to isolate and identify potential pathogens using previously described isolation methods. A randomized complete block design with six replicates per treatment was employed.

Successful pathogenicity was confirmed by the absence of disease symptoms in negative controls and the consistent appearance of symptoms in plants inoculated with the respective oomycete species.

## Results

### Phylogeny

In the analyses of multi-locus alignment 1 (*Globisporangium* species) (gene boundaries of ITS: 1–1253, *cox1*: 1254–1742 and *cox2*: 1743–2159), a total of 85 isolates belong to the genus *Globisporangium*, alongside an outgroup, were examined. The combined dataset (ITS + *cox1* + *cox2*) comprised 2159 characters, including alignment gaps, with 1132 variable characters (837 for ITS-rDNA, 169 for *cox1* and 126 for *cox2*) and 1027 constant characters (416 for ITS-rDNA, 320 for *cox1* and 291 for *cox2*).

**Fig. 1:**
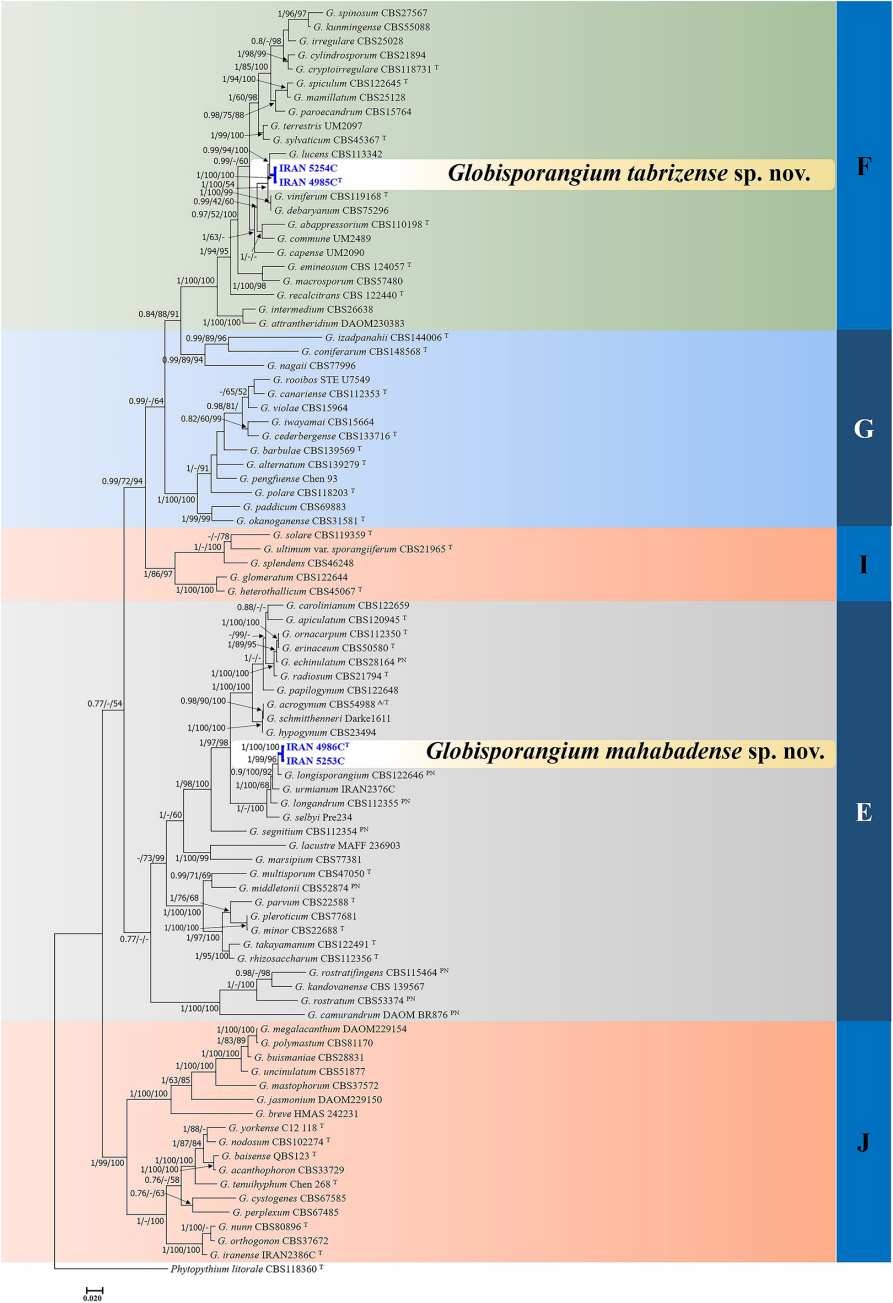
Phylogram generated from Bayesian inference analysis based on ITS-rDNA, *cox1*, and *cox2* sequence data for four examined strains and 85 reference strains belonging to *Globisporangium*. Numbers on the branches indicate posterior probabilities from Bayesian Inference as well as bootstrap support in Maximum Likelihood and Minimum Evolution, greater than 0.7/50%/50%, in the respective order. A dash indicates lower support for the presented topology or the possibility of an alternative topology. *Phytopythium litorale* type strain CBS118360 is used as outgroup. The obtained strains in this study are in blue. Clades E-G, I, and J, which were identified by Lévesque and de Cock in 2004^23^ within the *Pythium sensu lato*, are depicted on the right side of the figure. T and A/T indicate ex-type and authentic strains (respectively), probably used for original description and PN indicates authentic strains used for description in the monograph of van der Plaats-Niterink^21^.

Bayesian analysis confirmed the tree topology obtained from Minimum Evolution (ME) and Maximum Likelihood (ML) trees. While most Bayesian posterior probability values were consistent with bootstrap supports, the Bayesian tree was selected to represent the phylogeny and bootstrap values from ME and ML methods were incorporated for comparison (Fig. 1). In all three-locus phylogenetic trees (Bayesian, Maximum Likelihood and Minimum Evolution), the four *Globisporangium* isolates studied here were placed into two unique well supported clades, each representing a distinct species. Thus, the examined isolates IRAN 4986 C and IRAN 5253 C formed a robust monophyletic group with maximal support from Bayesian posterior probability (1) and bootstrap values (100/100 for ME/ML). Similarly, Iranian isolates IRAN 4985 C and IRAN 5254 C clustered together in a separate, strongly supported clade (Bayesian posterior probability 1; bootstrap 100/100 for ME/ML).

In the analyses of multi-locus alignment 2 (*Pythium* species) (gene boundaries of ITS: 1–976, *cox1*: 977–1526 and *cox2*: 1527–2016), a total of 61 isolates belonging to the genus *Pythium*, alongside an outgroup, were examined. The combined dataset (ITS + *cox1* + *cox2*) comprised 2016 characters, including alignment gaps, with 883 variable characters (506 for ITS-rDNA, 188 for *cox1* and 189 for *cox2*) and 1133 constant characters (470 for ITS-rDNA, 362 for *cox1* and 301 for *cox2*).

**Fig. 2:**
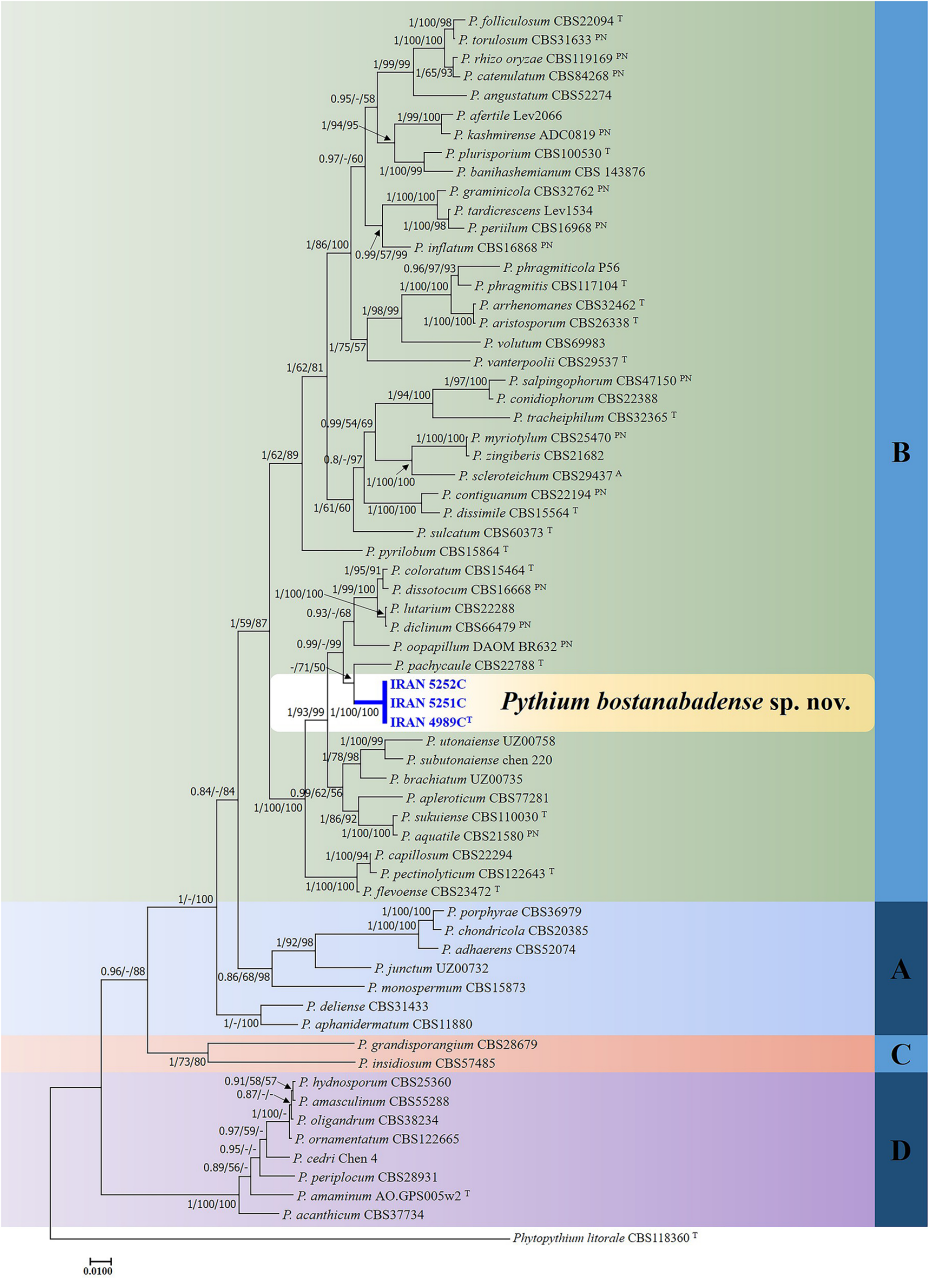
Phylogram generated from Bayesian inference analysis based on ITS-rDNA, *cox1* and *cox2* sequence data for three examined strains and 61 reference strains belonging to *Pythium*. Numbers on the branches indicate posterior probabilities from Bayesian Inference as well as bootstrap support in Maximum Likelihood and Minimum Evolution, greater than 0.7/50%/50%, in the respective order. A dash indicates lower support for the presented topology or the possibility of an alternative topology. *Phytopythium litorale* type strain CBS118360 is used as outgroup. The obtained strains in this study are in blue. Clades A-D which were identified by Lévesque and de Cock in 2004^23^ within the *Pythium sensu lato*, are depicted on the right side of the figure. T and A indicate ex-type and authentic strains (respectively), identified by the author of the species and PN indicates authentic strains used for description in the monograph of van der Plaats-Niterink^21^.

Phylogenetic analyses, employing Bayesian Inference, Maximum Likelihood and Minimum Evolution methods, converged on a consistent topological framework for the *Pythium* isolates examined. Robust Bayesian posterior probabilities and bootstrap support values decisively recognized the three *Pythium* strains (IRAN 4989 C, IRAN 5251 C, IRAN 5252 C) as a monophyletic clade, as evidenced by their clustering in a well-defined clade (Bayesian posterior probability 1; bootstrap 100/100 for ME/ML) (Fig. 2).

**Fig. 3:**
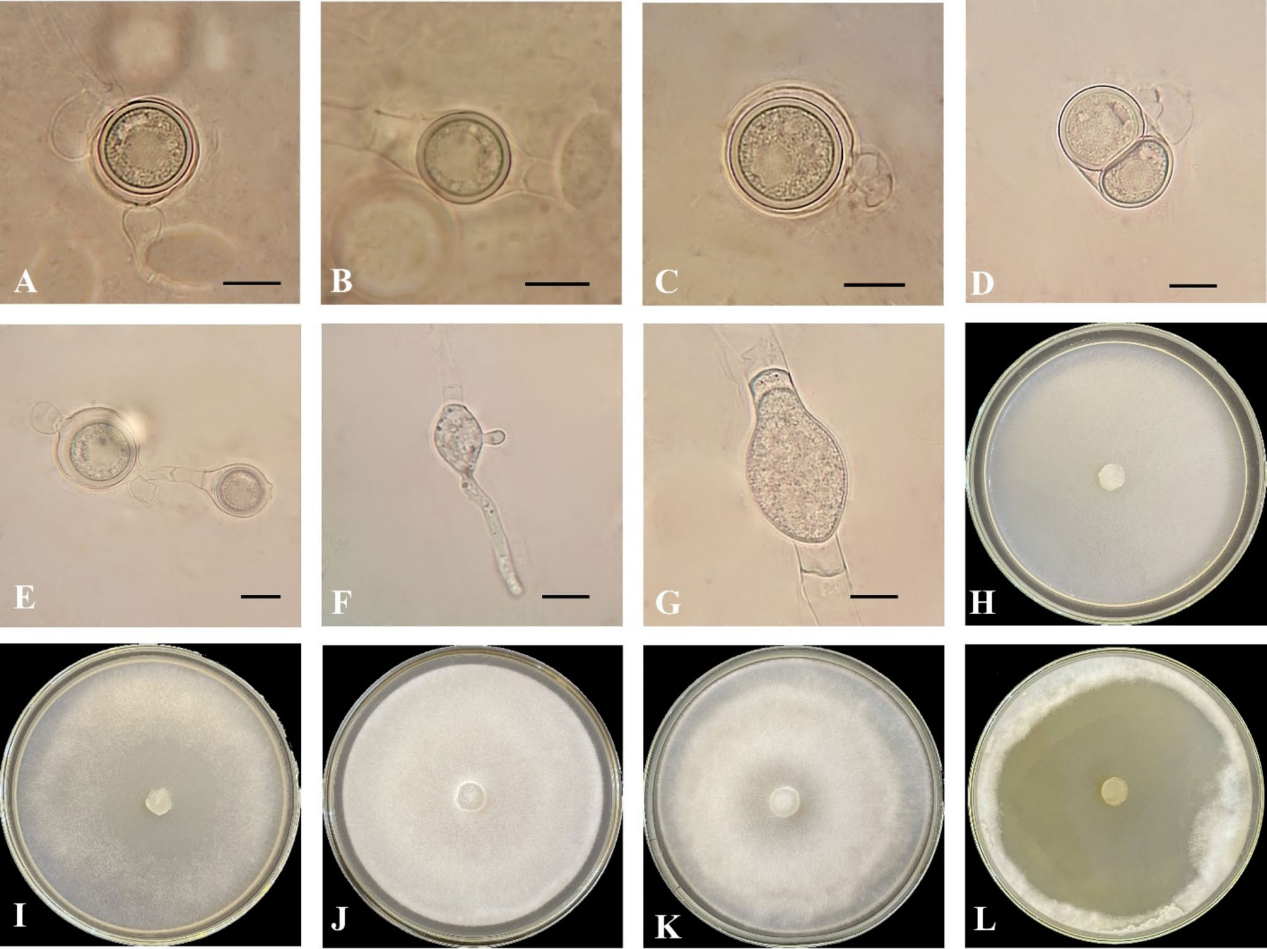
*Globisporangium tabrizense* isolate IRAN 4985 C. (**A**) oogonium with two monoclinous and diclinous antheridium. (**B**) intercalary oogonium and plerotic oospore. (**C**) oogonium with one antheridium. (**D**) oogonium with one antheridium and two oospores. (**E**) chain oogonium. (**F**) sporangium with Two discharge tubes. (**G**) intercalary sporangium. (H–L) colony on various media; (**H**) CMA. (**I**) PCA. (**J**) V8A. (**K**) PDA. (**L**) MEA. Scale bars: 10 μm.

In multi-locus analyses of both datasets, alignment 1 and alignment 2, the topologies of the single-locus phylogenies (Supplementary Figure S1) did not conflict with the respective three-loci phylogenies, confirming the reliability and accuracy of the inferred relationships among these isolates.

### Taxonomy

This study discovered new oomycete species in various aquatic environments in Iran. Four isolates of the genus *Globisporangium*, including two from roots of the grass species *Cynodon dactylon*, growing within the agricultural irrigation canals (IRAN 4985 C and IRAN 5254 C) and two from water surface algae in agricultural irrigation canals in Mahabad irrigation water (IRAN 4986 C and IRAN 5253 C), were determined as two new species: *Globisporangium tabrizense* sp. nov. and *G. mahabadense* sp. nov., respectively. Additionally, three isolates (IRAN 4989 C, IRAN 5251 C, and IRAN 5252 C) from water surface algae in agricultural pools were identified as a new species, *Pythium bostanabadense* sp. nov. (Supplementary Table S2). All species studied are characterized below.

***Globisporangium tabrizense* Ahadi**,** Chenari Bouket**,** Alizadeh**,** Masigol and Grossart sp. nov.**

Figure 3.

MycoBank: 854997.

**Typification**: IRAN. East Azarbaijan: Tabriz (Varanaq), from roots of the grass species *Cynodon dactylon*, growing within the agricultural irrigation canals, Oct 2022, *R. Ahadi* (**holotype** IRAN 18502 F). Ex-holotype culture IRAN 4985 C = AZFC-RAG178-5-1. GenBank: ITS = PQ037624; *cox1* = PQ031210; *cox2* = PQ031204.

**Etymology**: Referring to the city of Tabriz, in the vicinity of which the species was collected.

**Morphology**: The colony pattern on V8A, PCA, MEA and PDA appeared intermediate and radial pattern on CMA, with a daily growth rate at 25 °C on PCA recorded at 21 mm. The cardinal temperatures were noted as a minimum of 2 °C, an optimum of 25 °C and a maximum of 35 °C on PCA. The main hyphae were hyaline, aseptate and ranged from 2.5 to 8.5 μm in width. The sporangia were diverse, with globose sporangia appearing intercalary or terminal, measuring 9.5–26 μm in diameter (mean, 17 μm); lemon-shaped sporangia measuring 14.5–40 μm in length (mean, 21.5 μm) and 7–22 μm in width (mean, 12.5 μm), alongside filamentous sporangia. Zoospores were not observed. Oogonia were globose and smooth, appearing intercalary or terminal, with a diameter range of 13.5–22 μm (mean, 18.3 μm) rarely 2 in a chain and produced in single cultures. Antheridia were typically one, occasionally two per oogonium, diclinous or monoclinous. Oospores were either aplerotic or plerotic, with one, rarely two, per oogonium, measuring 12.2–22 μm in diameter (mean, 16.5 μm). The wall thickness ranged between 1.2 and 1.8 μm.

**Additional specimen examined**: IRAN. East Azarbaijan: Tabriz (Varanaq), from roots of the grass species *Cynodon dactylon*, growing within the agricultural irrigation canals, Oct 2022, *R. Ahadi.* culture (IRAN 5254 C = AZFC-RAG178-5-2). GenBank: ITS = PQ037625; *cox1* = PQ031211; *cox2* = PQ031203.

**Notes**: Phylogenetic analysis revealed a close phylogenetic relationship between *G. tabrizense* and *G. lucens*, followed by a slightly less close relationship with *G. viniferum* and *G. debaryanum*. The ex-type strain of *G. tabrizense* (IRAN 4985 C = AZFC-RAG178-5-1) exhibited 95% identity with *G. lucens* strain CBS113342 in the ITS region, and 99% identity in both *cox1* and *cox2*. However, it also displayed 19 nucleotide differences in ITS, one in *cox1*, and two in *cox2* compared to this strain. Blastn searches on NCBI GenBank indicated that the ITS sequence of *G. tabrizense* shared the highest similarity (99% identity, with seven nucleotide differences and two gaps) with *G. viniferum* isolate OPU 1675 (KU743395). Its *cox1* sequence was most similar (99%) to *G. lucens* CBS113342 (HQ708725), while the *cox2* sequence matched to 100% that of *G. sylvaticum* isolate PyTz77 (OK309797). The ex-type strain of *G. tabrizense* shared 97% identity in ITS, 99% in *cox1*, and 98% in *cox2* with *G. viniferum* voucher CBS119168, differing by 14 nucleotides in ITS, two in *cox1*, and six in *cox2*. Additionally, it shared 99% identity in ITS and *cox1*, and 98% in *cox2* with *G. debaryanum* CBS75296, but exhibited three nucleotide differences in ITS, two in *cox1*, and six in *cox2*. The examined loci consistently place *G. tabrizense* in a separate phylogenetic clade, confirming its status as a distinct species within *Globisporangium*.

*Globisporangium tabrizense* shares some morphological similarities with *G. lucens*^53^, *G. viniferum*^54^, and *G. debaryanum*^55^. However, distinct morphological features differentiate *G. tabrizense* from its closely related species. Compared to *G. lucens*, *G. tabrizense* has wider main hyphae, ranging from 2.5 to 8.5 μm, compared to 3.5–6.5 μm in *G. lucens*. Additionally, *G. tabrizense* lacks zoospores, while *G. lucens* produces zoospores. Furthermore, *G. tabrizense* produces both plerotic and aplerotic oospores, whereas *G. lucens* produces only aplerotic oospores. In terms of sporangium morphology, *G. tabrizense* exhibits greater diversity, including lemon-shaped and filamentous forms, while *G. lucens* primarily has globose or subglobose sporangia. When compared to *G. viniferum*, *G. tabrizense* again demonstrates a distinct sporangium morphology with the presence of lemon-shaped and filamentous forms. Moreover, *G. tabrizense* typically possesses one or two antheridia per oogonium, while *G. viniferum* can have up to five antheridia. Differentiating *G. tabrizense* from *G. debaryanum* is primarily based on the absence of zoospores and the production of plerotic oospores in *G. tabrizense*, while *G. debaryanum* produces only aplerotic oospores. Additionally, *G. tabrizense* often produces two oospores per oogonium, whereas *G. debaryanum* typically has only one. In summary, *G. tabrizense* is morphologically distinct from its congeners due to its unique combination of sporangia types, absence of zoospores, oospore characteristics, and antheridia number. These morphological differences highlight the taxonomic distinctiveness of *G. tabrizense* within the genus *Globisporangium* (Supplementary Table S3a and Figure S2).

***Globisporangium mahabadense* Ahadi**,** Chenari Bouket**,** Alizadeh**,** Masigol and Grossart sp. nov.**

Figure 4.

MycoBank: 852636.

**Typification**: IRAN. West Azarbaijan: Mahabad, from water surface algae in agricultural irrigation canals, Oct 2022, *R. Ahadi* (**holotype** IRAN 18503 F). Ex-holotype culture IRAN 4986 C = AZFC-RAG201-2-1. GenBank: ITS = PQ037626; *cox1* = PQ031213; *cox2* = PQ031206.

**Etymology**: Referring to the city, Mahabad, from which the species was collected.

**Morphology**: Colonies pattern on PDA was a rosette pattern, radial pattern on CMA, intermediate pattern on MEA and PCA, chrysanthemum pattern on V8A. Daily growth at 25 °C on PCA 9.5 mm. Cardinal temperatures were a minimum 5 °C, optimum 25 °C and maximum 30 °C on Potato Carrot Agar. Main hyphae hyaline, aseptate, 2.5–5 μm (mean, 4 μm) wide. Sporangia globose intercalary or terminal, 11–33 μm (mean, 25 μm) in diameter. Zoospores not observed. Oogonium is rarely formed, terminal and intercalary, smooth, globose, 14–23 μm (mean, 20 μm) in diameter and produced in a single culture. Antheridia one per oogonium, diclinous. Oospore globose or semi-globose, plerotic, one per oogonium, globose 16–23 μm (mean, 21 μm) in diameter and semi-globose 18–19.5 (mean, 19) length and 15–17 μm (mean, 16.5 μm) width. Wall thickness was between 1 and 3 μm.

**Additional specimen examined**: IRAN. West Azarbaijan, Mahabad, from water surface algae in agricultural irrigation canals, Oct 2022, *R. Ahadi.* culture (IRAN 5253 C = AZFC-RAG201-2-2). GenBank: ITS = PQ037627; *cox1* = PQ031212; *cox2* = PQ031205.

**Notes**: Phylogenetic analysis revealed a close relationship between *Globisporangium mahabadense* and *G. longisporangium*, followed by a slightly less close relationship with *G. urmianum*, *G. longandrum* and a slightly distant relationship with *G. selbyi*.

BLASTn searches on NCBI GenBank revealed that the ITS sequence of the *G. mahabadense* ex-type strain (IRAN 4986 C = AZFC-RAG201-2-1) exhibited the highest identity (99%) with the *G. bifurcatum* type strain F-91 (AY083935) and the *G. longisporangium* isolate CBS122646 (HQ643680), differing by two and eight nucleotides, respectively. Similarly, the *cox1* and *cox2* sequences of the ex-type strain shared 98% and 99% identity, respectively (with eight and five nucleotide differences), with *G. longisporangium* isolate CBS122646 (HQ708724). Comparison of *G. mahabadense* with *G. urmianum* IRAN2376 showed 98% ITS and 97% *cox1* identity, but with 12 nucleotide differences in the ITS region and 14 in the *cox1* region. Comparative analysis of *G. mahabadense* with *G. longandrum* CBS112355 showed 98% ITS, 96% *cox1*, and 98% *cox2* identity, with 12, 15, and 3 nucleotide differences, respectively. Finally, *G. mahabadense* shared 97% ITS and *cox1* identity with *G. selbyi* CBS 129,729, and 96% *cox2* identity. The two species differed by 19 nucleotides in ITS, 13 in *cox1*, and 17 in *cox2*. Based on ITS, *cox1*, and *cox2* sequence data, *G. mahabadense* can be reliably distinguished from all other *Globisporangium* species. The phylogenetic data robustly establishes *G. mahabadense* as a phylogenetically isolated species within the genus *Globisporangium*.

**Fig. 4:**
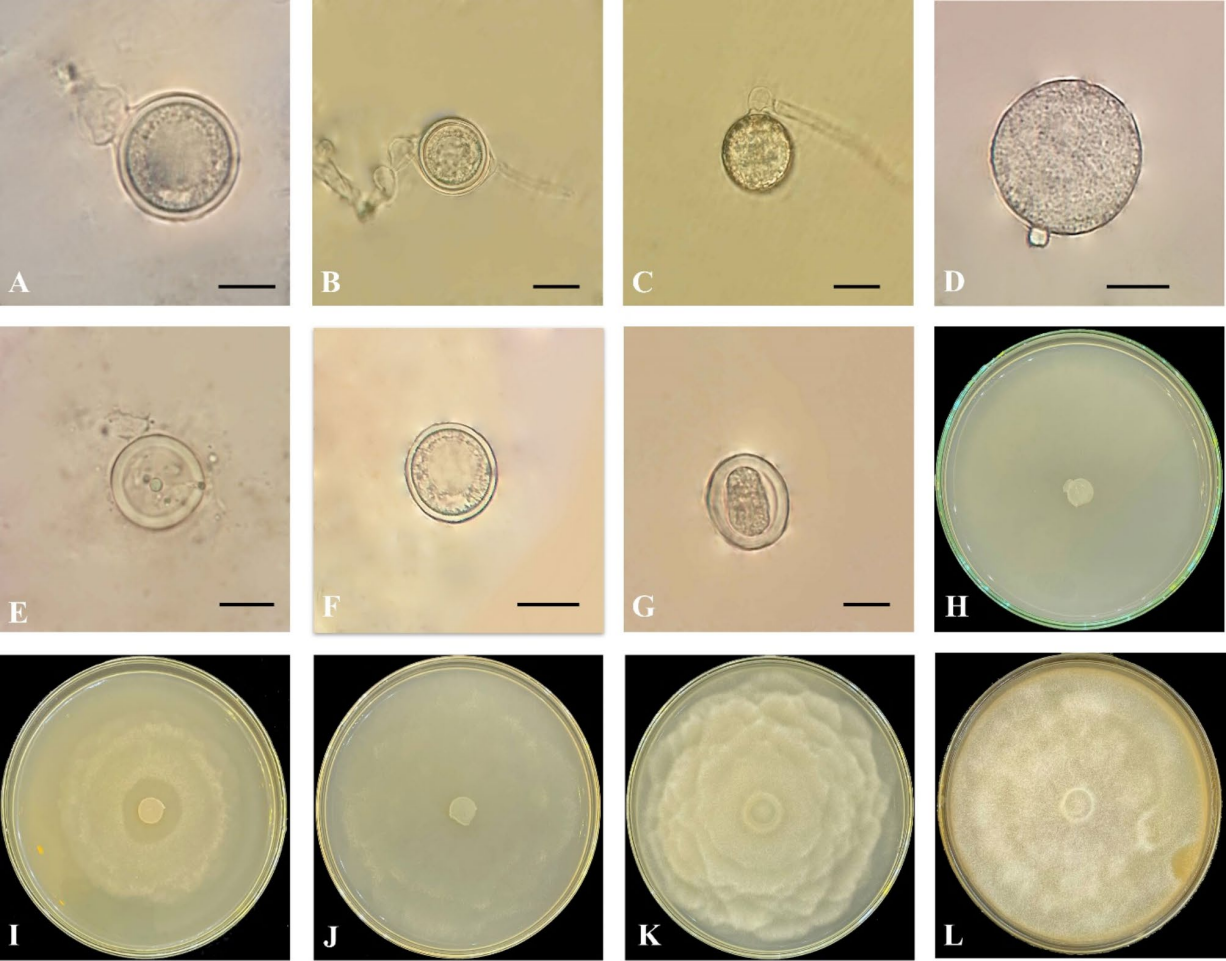
*Globisporangium mahabadense* sp. nov. isolate IRAN 4986 C. (**A**) terminal oogonium with one antheridium. (**B**) middle oogonium with one antheridium. (**C**) immature oogonium. (**D**) sporangium. (**E**) oogonium with attached antheridium and emptied oospore. (**F**) plerotic oospore. (**G**) plerotic oospore and semi-globose. (H–L) colony on various media; (**H**) CMA. (**I**) MEA. (**J**) PCA. (**K**) PDA. (**L**) colony on V8A. Scale bars: 10 μm.

*Globisporangium mahabadense* is morphologically distinct from its phylogenetically close species due to morphological characteristics. A detailed morphological comparison of *G. mahabadense*, *G. urmianum*, *G. longisporangium*^56^, and *G. longandrum*^57^ reveals distinct characteristics. *G. mahabadense* stands out with significantly narrower hyphae, measuring between 2.5 and 5 μm in width, compared to 6–8 μm in both *G. longisporangium* and *G. longandrum*. Additionally, hyphae of *G. mahabadense* are significantly thinner (2.5–5 μm) compared to those of *G. urmianum* (up to 7 μm). While *G. mahabadense* exclusively forms globose sporangia, *G. longisporangium* exhibits a broader range of shapes including cylindrical, peanut, and oval. *G. longandrum* primarily produces globose to elongated sporangia. In contrast, *G. urmianum* also shows a wider variety of sporangia shapes, including globose, subglobose, ellipsoidal, elongated, ovoid, or pyriform (Fig. 4). *G. mahabadense* oogonia are less frequent and generally smaller and more uniform in shape (globose, 14–23 μm diameter), compared to *G. urmianum* oogonia which can be globose or elongated (20–37 μm diameter) and more variable in size. Both species produce one or two antheridia per oogonium. In contrast, *G. longisporangium* commonly forms oogonia, often with 1–3 antheridia per oogonium. *G. longandrum* features more complex antheridial structures. A key distinguishing feature of *G. mahabadense* is the consistent formation of globose and semi-globose, plerotic oospores, whereas *G. urmianum* produces a wider range of oospore shapes, including globose, subglobose, and peanut-shaped, and can be either plerotic or aplerotic. *G. longisporangium* often has 1–3 oospores per oogonium, and *G. longandrum* typically forms 1–2 oospores. These morphological characteristics, including narrower hyphae, simpler sporangial shapes, consistent oospore formation, and unique antheridial structures, clearly differentiate *G. mahabadense* from its closely related species (Supplementary Table S3b).

**Fig. 5:**
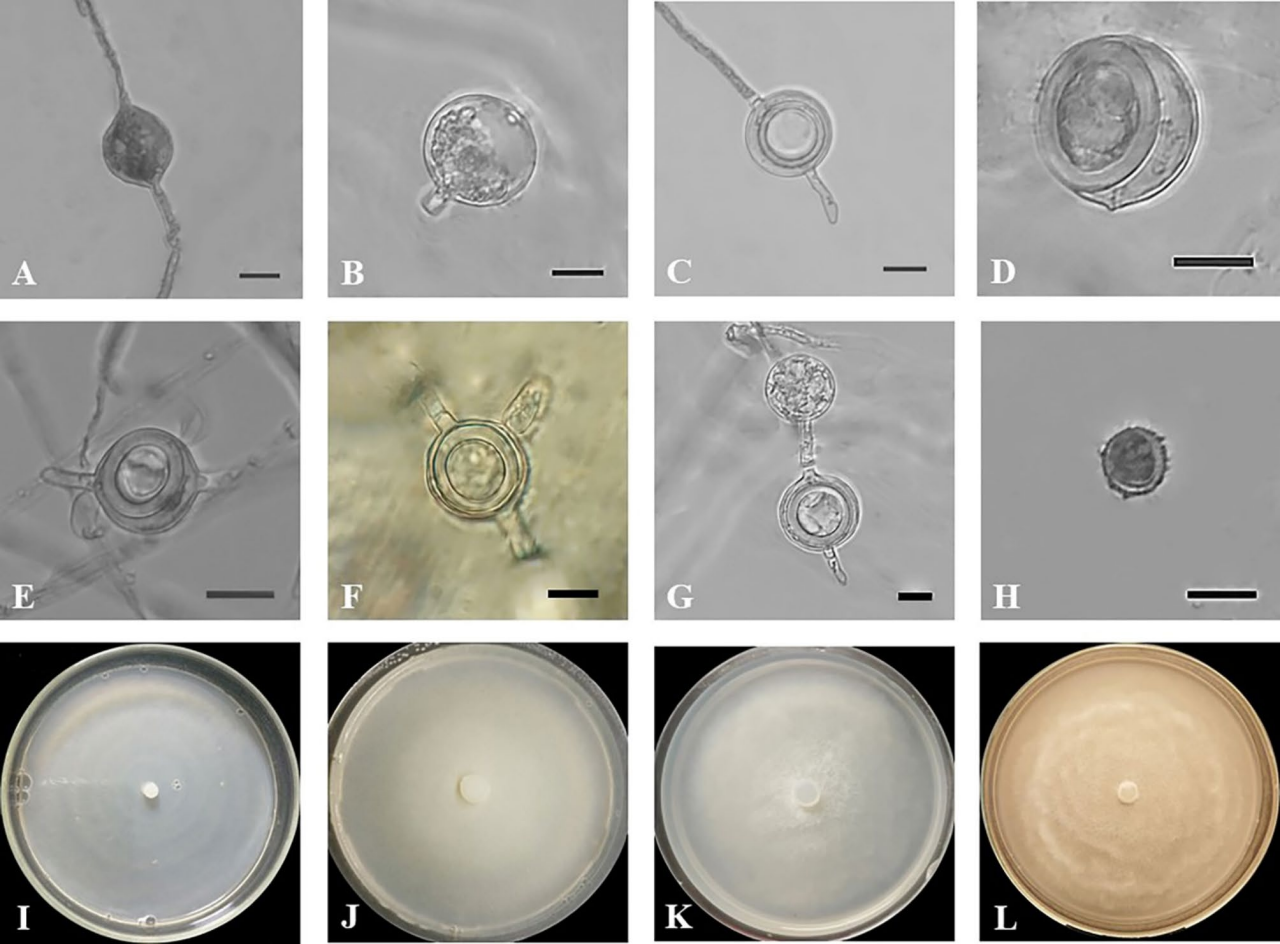
*Pythium bostanabadense* sp. nov. isolate IRAN 4989 C. (**A**) hyphae swelling. (**B**) sporangium. (**C**) aplerotic oospore with a papilla. (**D**) aplerotic oospore. (**E**) oogonium with two diclinous antheridia. (**F**) oogonium with one diclinous antheridium. (**G**) aplerotic oospores and sporangium chain. (**H**) zoospore. (I–L) colony on various media; (**I**) MEA. (**J**) PDA. (**K**) CMA. (**L**) V8A. Scale bars: 10 μm.

***Pythium bostanabadense *Ahadi**,** Chenari Bouket**,** Alizadeh**,** Masigol and Grossart sp. nov.**

Figure 5.

MycoBank: 852587.

**Typification**: IRAN. East Azarbaijan: Bostanabad, from water surface algae in agricultural pools, Oct 2022, *R. Ahadi* (**holotype** IRAN 18506 F). Ex-holotype culture IRAN 4989 C = AZFC-RAP159-1. GenBank: ITS = PQ037628; *cox1* = PQ031214; *cox2* = PQ031207.

**Etymology**: Named after the location, Bostanabad city, where the type was isolated.

**Morphology**: The colony on V8A displayed a rosette pattern, chrysanthemum pattern on CMA, unique growth on MEA and uniform on PDA. Daily growth on PCA measured 17.5 mm at 25 °C. Cardinal temperatures were recorded as a minimum of 2 °C, optimum at 25 °C and maximum at 35 °C on PDA. The main hyphae were hyaline, aseptate, 1.4–4 μm (mean, 3 μm) wide. Globose sporangia were either intercalary or terminal, measuring 12–22.5 μm (mean, 17.6 μm) in diameter; spindle hyphae showed swelling, with lengths ranging from 12 to 17.5 μm and widths from 16 to 20 μm. Zoospores were 7.5–12 μm in diameter. Oogonia were globose, smooth, intercalary or terminal, with 17.5–24 μm (mean, 19.5 μm) in diameter and were produced singularly in culture. Antheridia were usually 1, occasionally 2 per oogonium, diclinous. Oospores were either aplerotic or plerotic, one per oogonium, with diameters ranging from 14.5 to 20 μm. Wall thickness was between 1.5 and 3.5 μm.

**Additional specimens examined**: IRAN. East Azarbaijan: Bostanabad, from water surface algae in agricultural pools, Oct 2022, *R. Ahadi.* culture (IRAN 5251 C = AZFC-RAP159-2). GenBank: ITS = PQ037629; *cox1* = PQ031215; *cox2* = PQ031209; IRAN. East Azarbaijan: Bostanabad, from algae in pool, Oct 2022, R. Ahadi. culture (IRAN 5252 C = AZFC-RAP159-3). GenBank: ITS = PQ037630; *cox1* = PQ031216; *cox2* = PQ031208.

**Notes**: Phylogenetic analysis revealed that *P. pachycaule* is the closest relative of *P. bostanabadense*. The ex-type strain of *P. bostanabadense* (IRAN 4989 C = AZFC-RAP159-1) exhibited 97% identity with *P. pachycaule* strain CBS22788 in the ITS region, and 96% identity in both *cox1* and *cox2*. However, it also displayed 17 nucleotide differences in ITS, 20 in *cox1*, and 19 in *cox2* compared to this strain. Blastn searches on NCBI GenBank indicated that the ITS sequence of the *P. bostanabadense* ex-type strain exhibited 99% identity (with four nucleotide differences) to *Pythium* sp. isolate JN-6 from Lake Constance, Germany (DQ232767)^15^. The *cox1* sequence showed 99% identity (with six nucleotide differences and three single nucleotide gaps) to *Pythium* sp. isolate C12-9 from Lake Constance, Germany (KT692750)^58^. The *cox2* sequence showed 97% identity (with 15 nucleotide differences) to *P. dissotocum* strain KNU2301TP from South Korea (OQ700848). Phylogenetic analyses based on the examined loci (ITS, *cox1*, and *cox2*), unequivocally support *P. bostanabadense* as a distinct species within the genus *Pythium*.

*Pythium bostanabadense* and *P. pachycaule*^53^ share several morphological similarities as members of the genus *Pythium*. Both species exhibit globose oogonia, aplerotic oospores, and diclinous antheridia. However, distinct differences separate these two species. *P. bostanabadense* possesses narrower main hyphae (1.4–4 μm) compared to the broader hyphae (4–10 μm) of *P. pachycaule*. While *P. pachycaule* is characterized by filamentous sporangia, *P. bostanabadense* displays a combination of globose and spindle-shaped sporangia. Additionally, *P. bostanabadense* lacks the prominent neck observed on the oogonia of *P. pachycaule*. Oogonia size also differs, with *P. bostanabadense* having smaller oogonia (17.5–24 μm) compared to the larger oogonia (24–34 μm) of *P. pachycaule*. Furthermore, *P. bostanabadense* typically produces only one antheridium per oogonium, whereas *P. pachycaule* commonly has one to three antheridia. Finally, the absence of spindle-shaped oospores in *P. bostanabadense* contrasts with the occasional presence of such oospores in *P. pachycaule*. These combined morphological characteristics clearly differentiate *P. bostanabadense* from *P. pachycaule* (Supplementary Table S3c).

## Pathogenicity

**Fig. 6:**
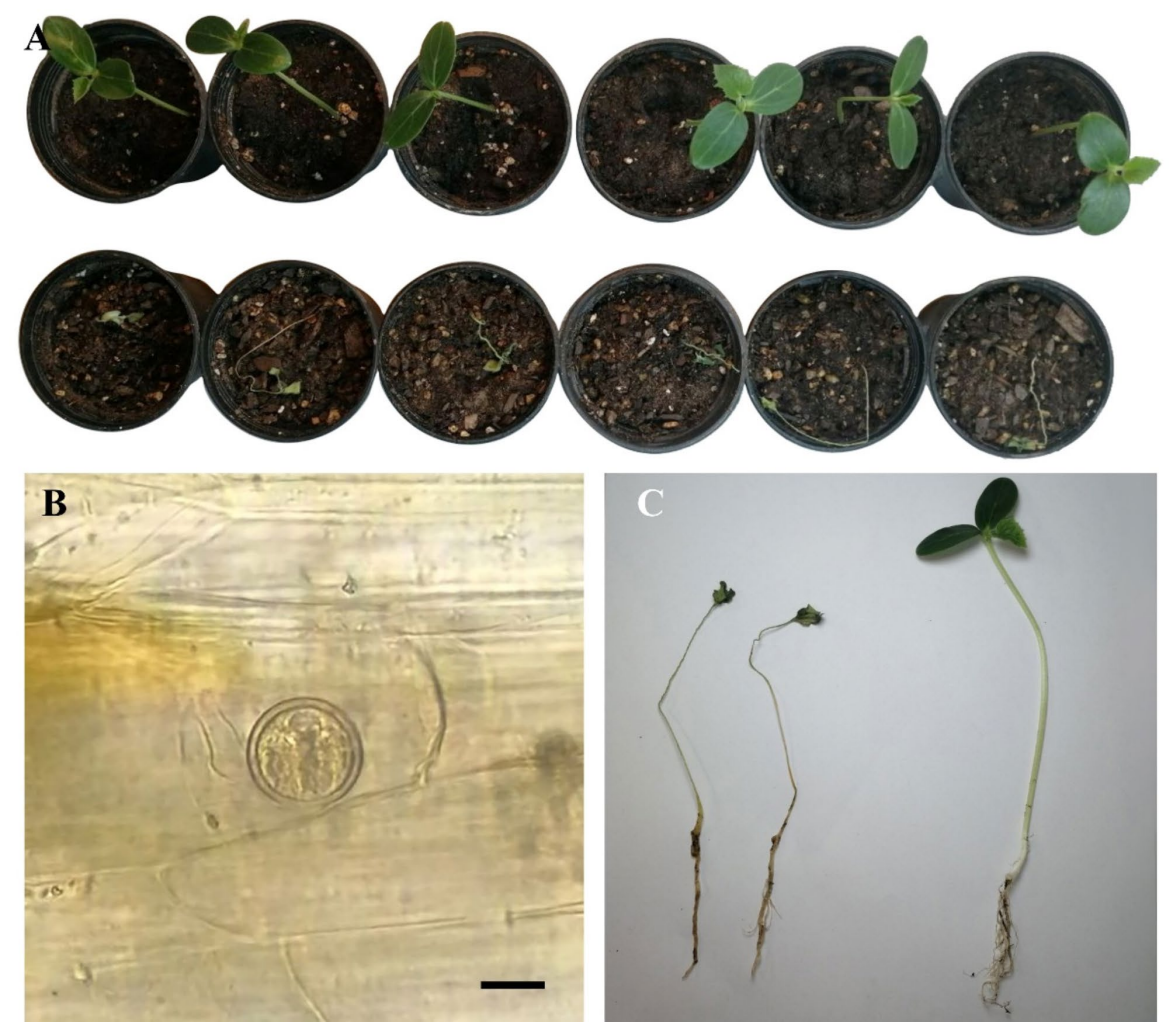
The pathogenicity test for *Globisporangium tabrizense* (isolate IRAN 4985 C) on cucumber plant after 14 days. (**A**) The up row of control pots and the bottom row of pots inoculated with *G. tabrizense*. (**B**) oogonium formed inside cucumber collar. (**C**) Control plant on the right and plants on the left inoculated with *G. tabrizense*. Scale bar: 10 μm.

**Fig. 7:**
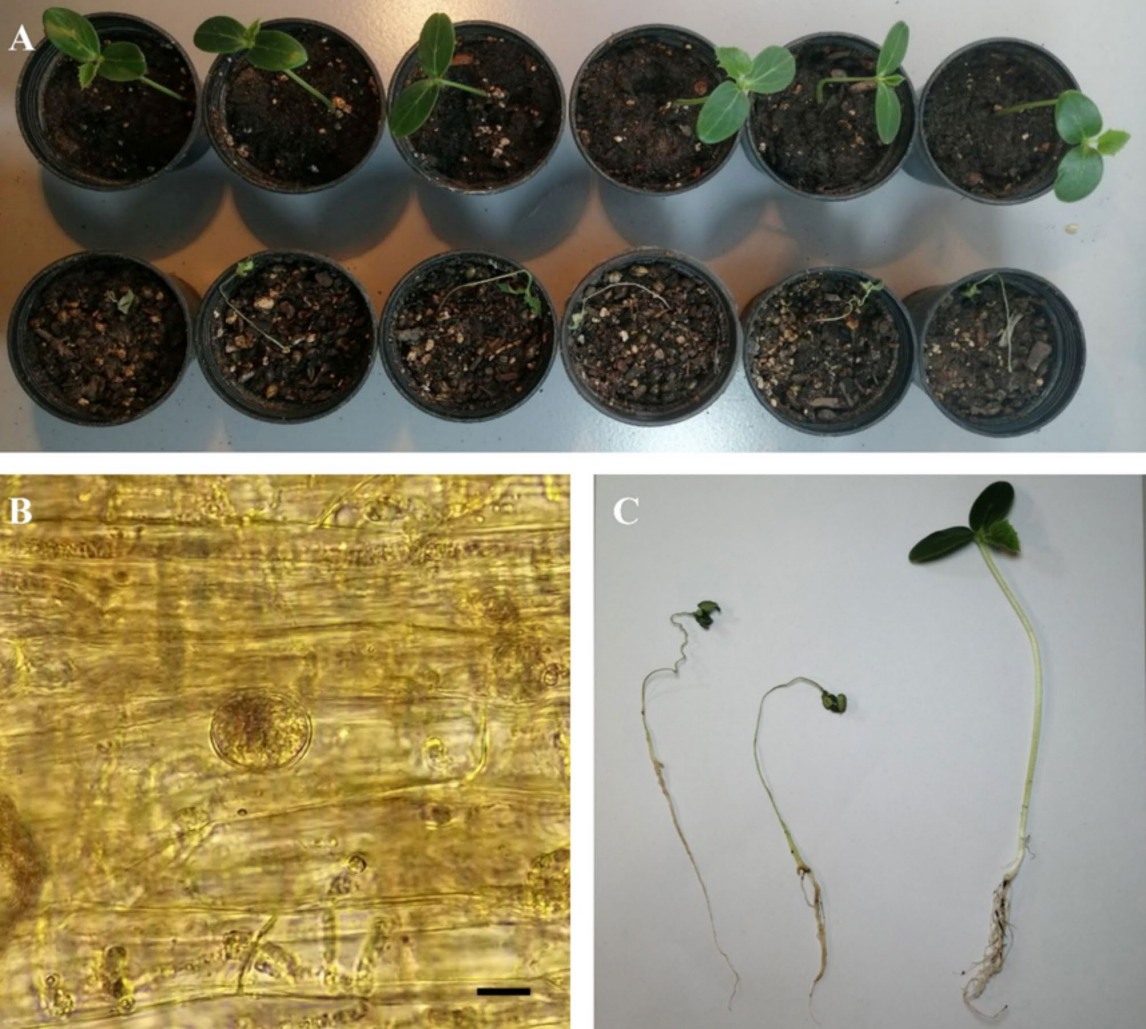
The pathogenicity test on cucumber plant for *Globisporangium mahabadense* sp. nov. (isolate IRAN 4986 C) on cucumber plant after 14 days. (**A**) The up row of control pots and the bottom row of pots inoculated with *G. mahabadense*. (**B**) Oospore formed inside cucumber collar. (**C**) Control plant on the right and plants on the left inoculated with *G. mahabadense*. Scale bar: 10 μm.

**Fig. 8:**
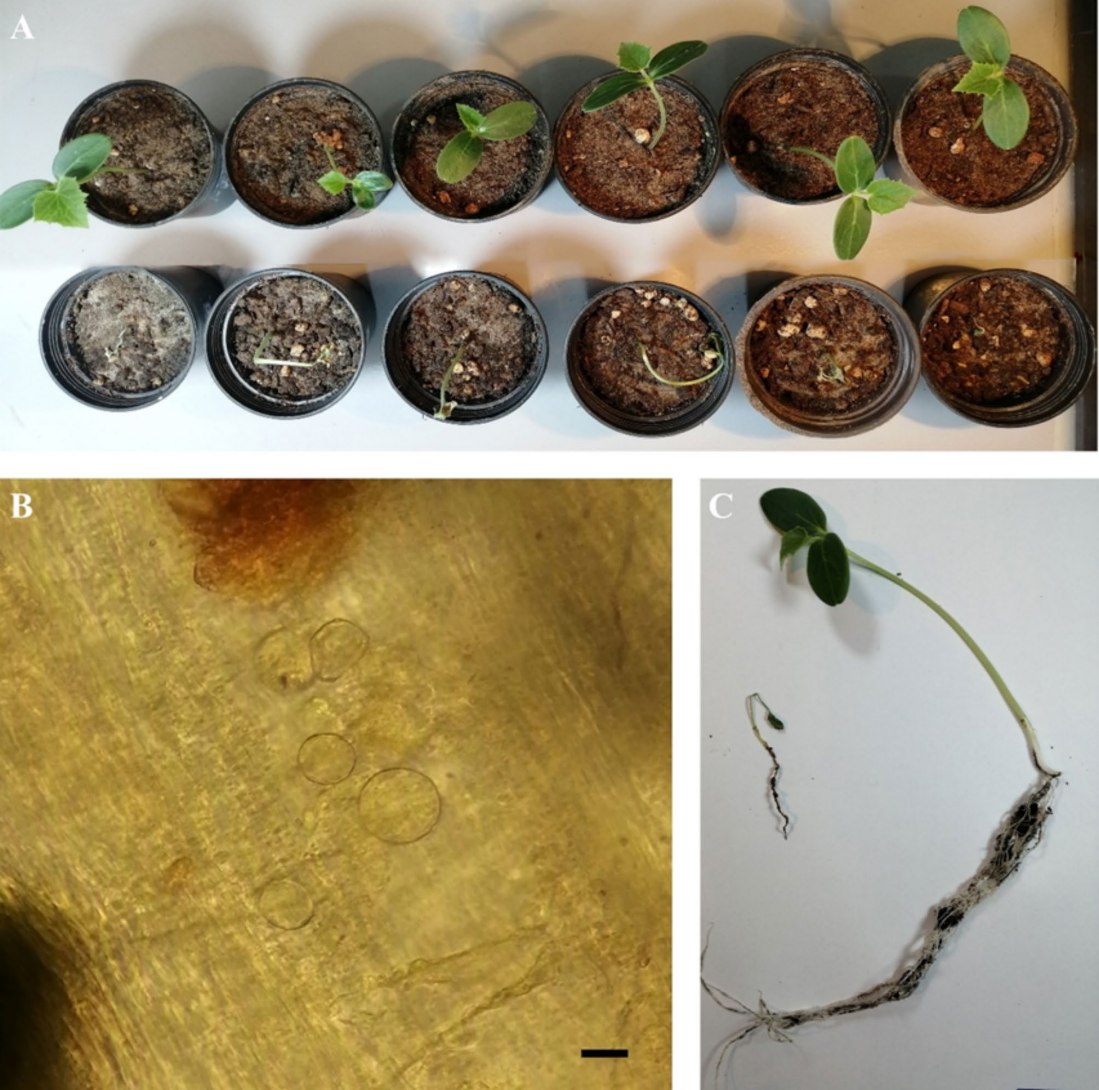
The pathogenicity test on cucumber plant for *Pythium bostanabadense* sp. nov. (isolate IRAN 4989 C) on cucumber plant after 14 days. (**A**) the up row of control pots and the bottom row of pots inoculated with the species. (**B**) sporangia formed inside cucumber collar. (**C**) control plant on the right and plant on the left inoculated with *P. bostanabadense* sp. nov. Scale bar: 10 μm.

Pathogenicity experiments confirmed the pathogenicity of all examined isolates, including *Globisporangium tabrizense* sp. nov. (IRAN 4985 C), *G. mahabadense* sp. nov. (IRAN 4986 C), and *Pythium bostanabadense* sp. nov. (IRAN 4989 C), resulting in crown and root rot and subsequent seedling death (Figs. 6, 7 and 8). All inoculated seedlings developed characteristic water-soaked lesions at the crown, leading to crown and root rot and eventual plant collapse within two weeks.

Three days post-sowing (dps), seedlings emerged normally. However, by 5 dps, initial symptoms of water-soaked lesions appeared on the crown, accompanied by incipient wilting. These symptoms intensified by 7 dps, with lesion coalescence, stem browning, and tissue decay extending to the root system. Control plants (negative control) remained symptomless throughout and they had much more root and stem growth compared to the positive treatments. Microscopic examination of infected tissue revealed sporangia and oospores of all three tested isolates. Furthermore, successful re-isolation of the original isolates from infected plants (100%) and their subsequent identification through detailed microscopy and morphometry fulfilled Koch’s postulates, unequivocally confirming their pathogenicity.

## Discussion

In this study, three novel species namely *Globisporangium tabrizense* sp. nov., *G. mahabadense* sp. nov., and *Pythium bostanabadense* sp. nov. were described. The identification of these species was based on a combination of multi-gene phylogenetic analysis utilizing nuclear rDNA ITS1–5.8 S–ITS2 (ITS), along with partial sequences of the cytochrome *c* oxidase subunit I and II (*cox1* and *cox2*), as well as morphological assessments. Our research is among the first to explore the diversity of *Pythium s.l.* in Iranian freshwater ecosystems, raising important questions about their ecological roles in non-terrestrial environments.

The selection of the ITS, *cox1*, *cox2* loci for phylogenetic clarification of the newly described species was guided by previous studies^59–64^ and the current limitations in available sequence data. While *cox2* has been widely used in oomycete phylogeny due to its high sequence variability and suitability for barcoding^62,65–69^, a comprehensive dataset for all described *Globisporangium* and *Pythium* species is still lacking. In contrast, *cox1*, when used alongside ITS, has been suggested as an effective barcode for oomycetes^70^. However, the amplification success of *cox1* can vary significantly among different oomycete lineages. These considerations prompted us to utilize all three loci (ITS, *cox1*, and *cox2*) in our analysis. Our phylogenetic analysis demonstrated that while all three genomic regions (ITS, *cox1*, and *cox2*) successfully resolved the unique phylogenetic positions of the newly identified species (Supplementary Figure S1), their power in distinguishing between these novel taxa and their closely related congeners within *Globisporangium* and *Pythium* varied. In other words, the performance of each genomic region to reveal interspecific genetic diversity differed (Supplementary Table S4). These findings emphasize the importance of employing a multi-gene approach in oomycete phylogeny to thoroughly capture genetic diversity and accurately delineate species boundaries.

In this study, phylogenetic analysis effectively distinguished *Globisporangium* and *Pythium* species into separate clades: Clades E-G, I, and J for *Globisporangium*, and Clades A-D for *Pythium*. This classification aligns with the clades identified by Lévesque and De Cock^23^ for *Pythium s.l.*, which were later confirmed by Robideau et al.^70^ and Nguyen et al.^71^ through phylogenomic analysis. Our findings indicate that species within Clade A can be further subdivided into two groups, aligning with the results of Salmaninezhad et al.^72^ and Rezaei et al.^73^. This divergence may stem from differences in the genomic regions analyzed in these studies compared to those used by Lévesque and De Cock^23^ and Nguyen et al.^71^.

The isolation of *P. bostanabadense* sp. nov., and *G. mahabadense* sp. nov. from the algal mats is in line with several other studies in which several *Pythium* species (*P. marinum* and *P. porphyrae*) has repeatedly considered as an algal pathogen from freshwater and marine environments^74,75^. More recently, *P. chondricola* De Cock has been shown to infect the blade of the red algae *Pyropia yezoensis* (Rhodophyta) in Korea and China, causing a disease namely red rot disease^76–78^. Similarly, *P. porphyrae* M. Takah. & M. Sasaki (as a phylogenetically close relative of *P. chondricola*) was shown to be pathogenic against another red algae *Pyropia plicata* from New Zealand^79^. Additionally, *Pythium* sp. strains (closely related to *P. dissotocum*) infected *Ulva* species (green alga, Chlorophyta) under low salinity levels^80^. Although the algal mats, from which *P. bostanabadense* sp. nov., and *G. mahabadense* sp. nov. were isolated, didn’t show any disease symptoms, we cannot rule out their pathogenic capabilities. Therefore, pathogenicity test(s) is necessary to validate the possibility of their pathogenicity against the associated algae. Another possibility would be that *P. bostanabadense* sp. nov., and *G. mahabadense* sp. nov. are terrestrial associated (plant/animal pathogens or saprophytes), but have been passively ended up in water via mainly plant litter and thereafter started colonizing the algal mats. The fact that their pathogenicity test was positive on cucumber plant might point out the notion that these *Pythium* species could be plant pathogens but using water as a vector simply for survival and dissemination. This notion has been supported by the occasional isolation of several plant pathogen *Pythium* taxa (e.g., *Pythium aphanidermatum*^81^ (Edson) Fitzp. and *Pythium myriotylum*^82^ Drechsler) from various aquatic environments^83,84^.

Cucumber (*Cucumis sativus* L.) was selected as a host plant to evaluate the pathogenic potential of the newly described species in this study. This plant is a highly susceptible host to a diverse range of oomycete pathogens, including *Globisporangium* and *Pythium* species^21,47–50^. These pathogens are notorious for causing damping-off, crown rot, and root rot. Well-known examples include *G. ultimum* var. *ultimum*^47^, *G. sylvaticum*, *G. irregulare*, *G. spinosum*^48^ *P. aphanidermatum* and *P. myriotylum*^21,49,50^. This established host-pathogen relationship made cucumber an ideal model system for evaluating the pathogenicity of our newly identified *Globisporangium* and *Pythium* species. Furthermore, cucumber is a crucial economic crop in Iran, often cultivated near rivers and other water bodies. Using cucumber as a host allowed us to directly assess the potential threat these oomycetes pose to agricultural production, particularly in regions relying on irrigation water from rivers and agricultural pools. Although not strictly aquatic, cucumber plants are frequently exposed to aquatic environments through irrigation and flooding. By employing cucumber as a host, we investigated the potential for oomycetes originating from aquatic ecosystems to infect and colonize terrestrial plants. Our pathogenicity tests revealed that all three newly identified species possess the ability to infect cucumber. These findings contribute significantly to our understanding of the ecological role oomycetes play in the transition from aquatic to terrestrial environments.

Although our results are important in terms of exploring hidden diversity of *Pythium s.l.* from freshwater ecosystems, one should be cautious when discussing the ecological relevance and implications of such taxonomy-based investigations. Cautionary steps are required as these studies are culture-dependent which often isolate *Pythium* taxa accidentally and also miss less abundant and slow-growing ones^85^. In our case, *Pythium* species associated with algal and root substrates could be very diverse but not isolated by culture-dependent techniques due to their very low efficiency. Therefore, we suggest that culture-based techniques to be accompanied by culture-independent approaches, e.g. metabarcoding, which minimize the abovementioned challenges and make the ecological interpretation of oomycete diversity composition more relevant^13^. So far, most *Pythium*-related culture-independent studies have conducted on terrestrial ecosystems, highlighting the knowledge gap regarding their hidden diversity and ecological contributions in aquatic ecosystems. In only one case, the metabarcoding approach was used to understand oomycete diversity in recycled irrigation water in a container nursery^13^. The study showed 57 and 36% of all OTUs detected from respectively filtration and leaf baiting techniques are assigned to either *Pythium sensu stricto* or *Phytopythium* with *Pythium chondricola*-complex and *Pythium monospermum-*like taxa as the most common species. However, it still needs to be investigated what ecological functions these *Pythium sensu lato* might have in semi-natural aquatic environments.

The findings of this study significantly contributed to the expanding knowledge of oomycete diversity, emphasizing their remarkable variety in aquatic environments and underscoring the importance of conducting biodiversity assessments in these ecosystems. Recent investigations by numerous researchers^86^, along with the results of this study, indicate that the true diversity of oomycetes has been largely underestimated. This is particularly concerning given that our understanding of oomycete diversity is predominantly focused on economically significant plant pathogens, leaving a substantial gap in our knowledge regarding the true diversity of aquatic oomycetes^87^. Considering the undiscovered diversity of oomycetes in aquatic environments, coupled with their ability to spread rapidly, especially through irrigation water, pathogenic oomycetes pose a significant threat to horticulture, forestry, agriculture, and aquaculture^88,89^.

Consequently, conducting biodiversity studies of oomycetes in various aquatic habitats is crucial for identifying oomycete plant pathogens that jeopardize agricultural practices and for developing effective disease management strategies.

## Conclusion

In this study, three novel species: *Globisporangium mahabadense* sp. nov., *G. tabrizense* sp. nov., and *Pythium bostanabadense* sp. nov. were described through a polyphasic approach based on combination of multi-gene phylogeny and morphology. Our findings, among the first to explore *Pythium sensu lato* diversity in Iranian freshwater ecosystems, raise questions about their ecological roles in non-terrestrial environments. The isolation of these species from algal mats, similar to known algal pathogens, suggests potential pathogenicity, though this remains to be tested. The positive pathogenicity test on cucumber plants indicates that these species might use water as a vector for plant infection. To fully understand their ecological relevance, culture-dependent techniques should be complemented with culture-independent approaches like metabarcoding, which can more accurately capture the diversity and ecological roles of these oomycetes in aquatic ecosystems.

## Electronic supplementary material

Below is the link to the electronic supplementary material.

Supplementary Material 1

Supplementary Material 2

Supplementary Material 3

Supplementary Material 4

Supplementary Material 5

Supplementary Material 6

## Data Availability

The datasets used and/or analyzed during the current study are available from the corresponding authors upon reasonable request.
