# Supplementary material for: Globisporangium tabrizense sp. nov., Globisporangium mahabadense sp. nov., and Pythium bostanabadense sp. nov. (Oomycota), three new species from Iranian aquatic environments
Source: Sci Rep. 2024 Dec 30;14:31701. doi: 10.1038/s41598-024-81651-0 (PMC11686014; doi:10.1038/s41598-024-81651-0)
Supplement: Supplementary file 1 — Supplementary Material 1 [file 41598_2024_81651_MOESM1_ESM.pdf]

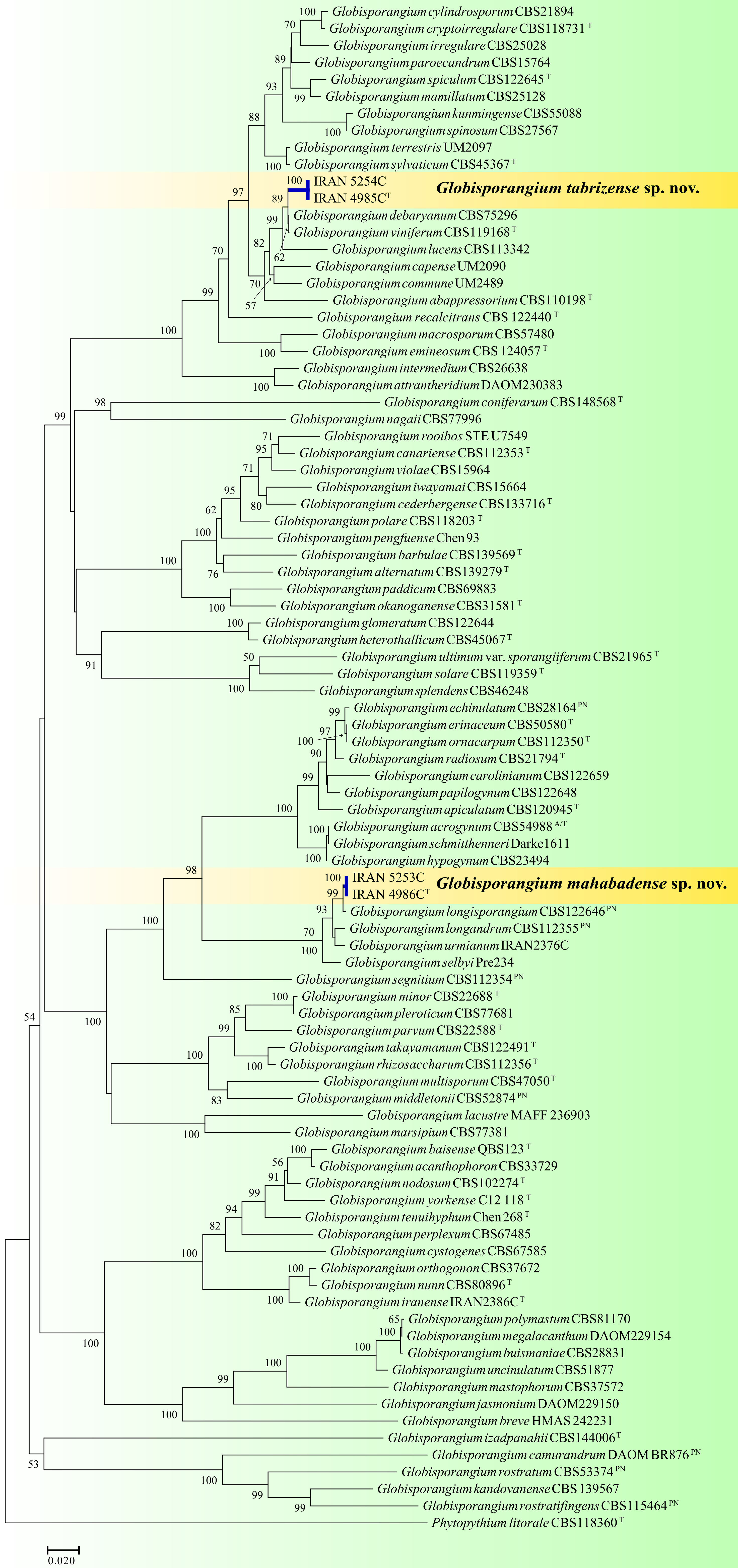

Figure 1. Minimum Evolution tree (ME) based on **ITS-rDNA** sequence data for four examined strains and reference strains belonging to ***Globisporangium***. Numbers on the branches indicate bootstrap support in Minimum Evolution greater than 50%. *Phytopythium litorale* type strain CBS118360 is used as outgroup. T indicates extype strains; A/T authentic strain, probably used for original description and PN indicates authentic strains used for description in the monograph of van der Plaats-Niterink[21].

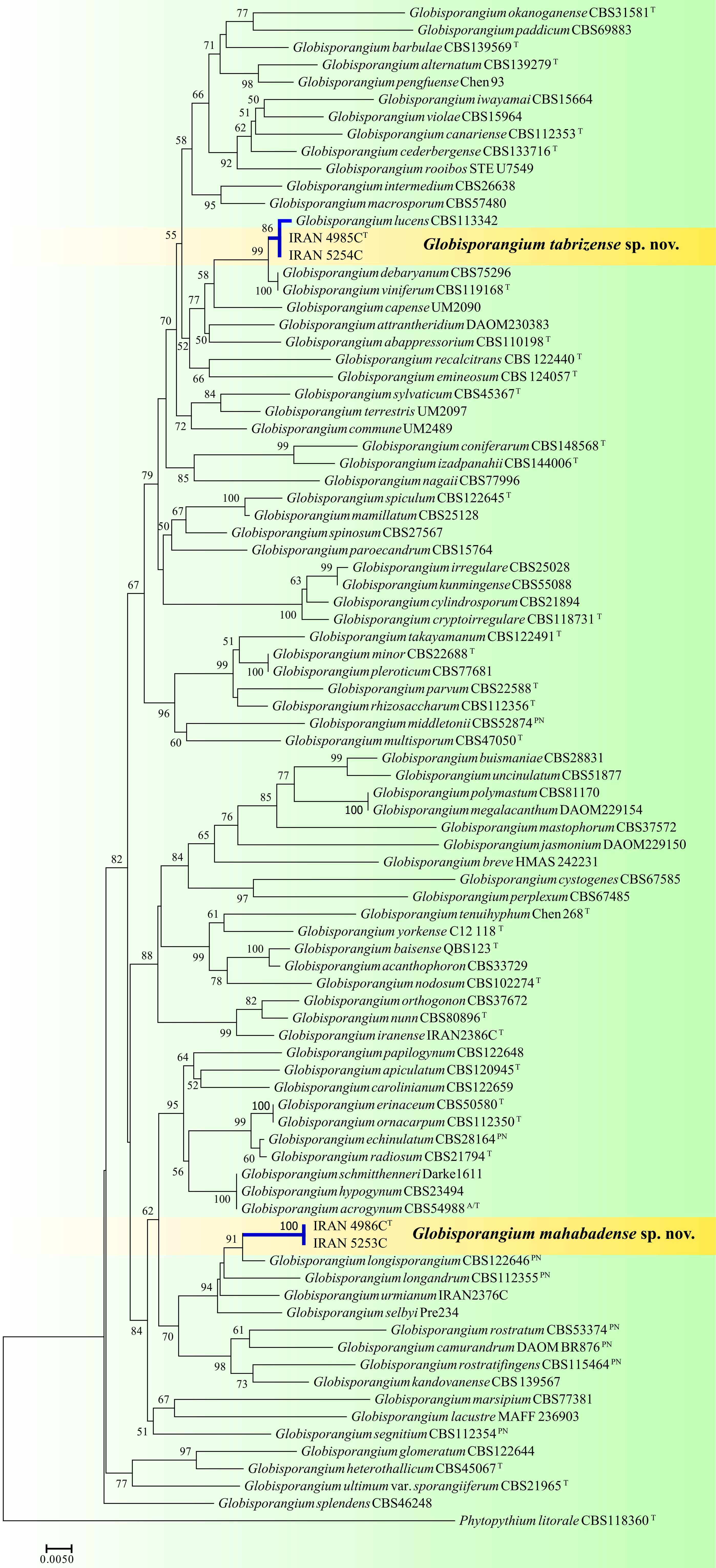

Figure 2. Minimum Evolution tree (ME) based on ***cox1*** sequence data for four examined strains and reference strains belonging to ***Globisporangium***. Numbers on the branches indicate bootstrap support in Minimum Evolution greater than 50%. *Phytopythium litorale* type strain CBS118360 is used as outgroup. T indicates ex-type strains; A/T authentic strain, probably used for original description and PN indicates authentic strains used for description in the monograph of van der Plaats-Niterink[21].

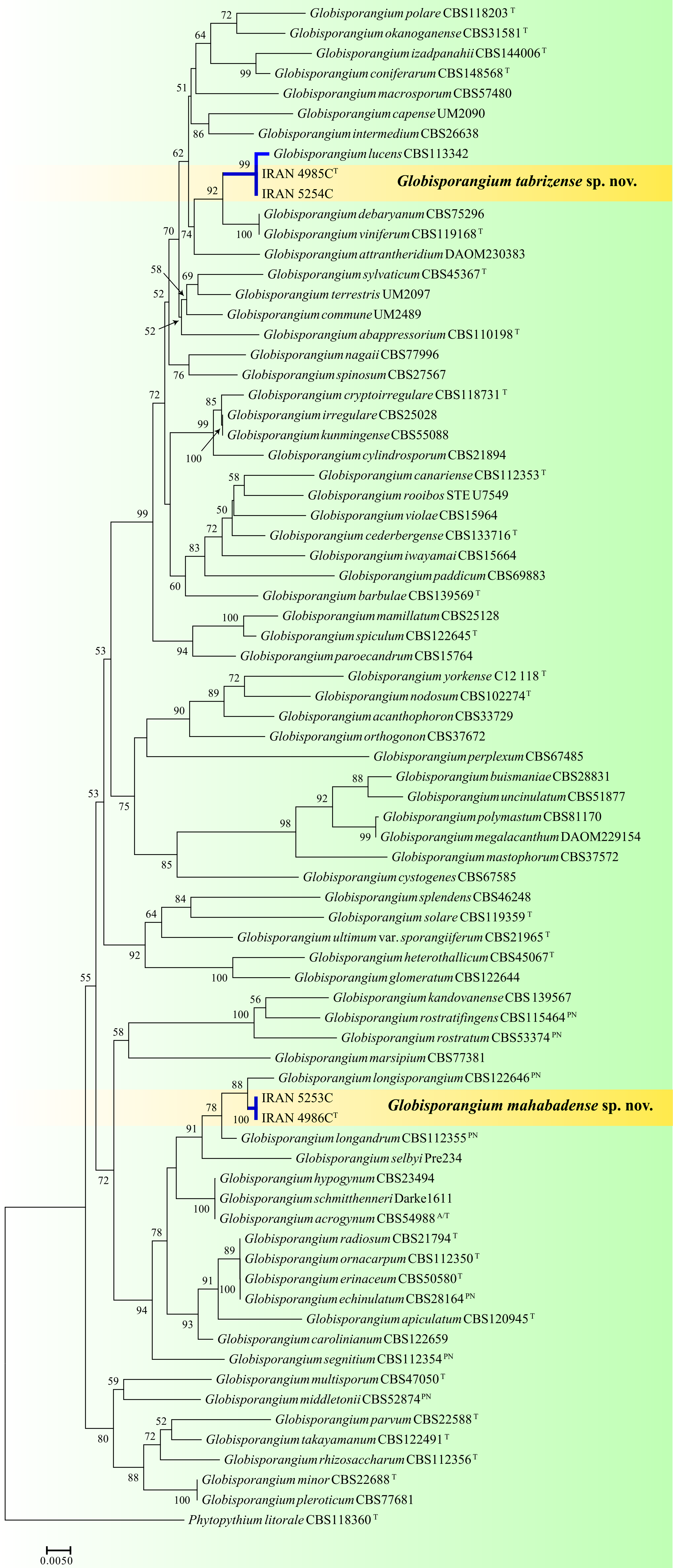

Figure 3. Minimum Evolution tree (ME) based on ***cox2*** sequence data for four examined strains and reference strains belonging to ***Globisporangium***. Numbers on the branches indicate bootstrap support in Minimum Evolution greater than 50%. *Phytopythium litorale* type strain CBS118360 is used as outgroup. T indicates ex-type strains; A/T authentic strain, probably used for original description and PN indicates authentic strains used for description in the monograph of van der Plaats-Niterink[21].

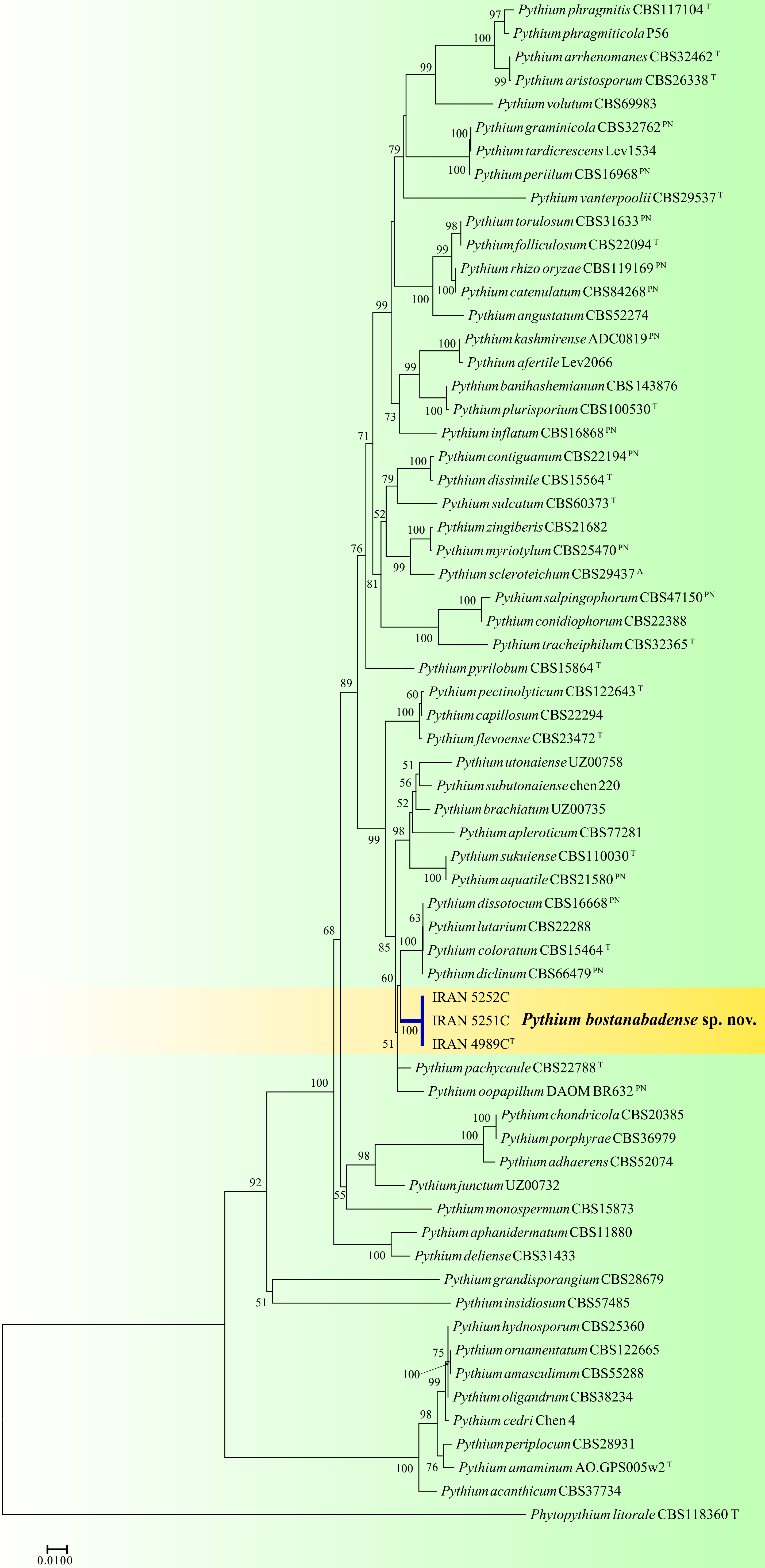

Figure 4. Minimum Evolution tree (ME) based on **ITS-rDNA** sequence data for three examined strains and reference strains belonging to ***Pythium***. Numbers on the branches indicate bootstrap support in Minimum Evolution greater than 50%. *Phytopythium litorale* type strain CBS118360 is used as outgroup. T indicates ex-type strains; A authentic strain, identified by the author of the species and PN indicates authentic strains used for description in the monograph of van der Plaats-Niterink[21].

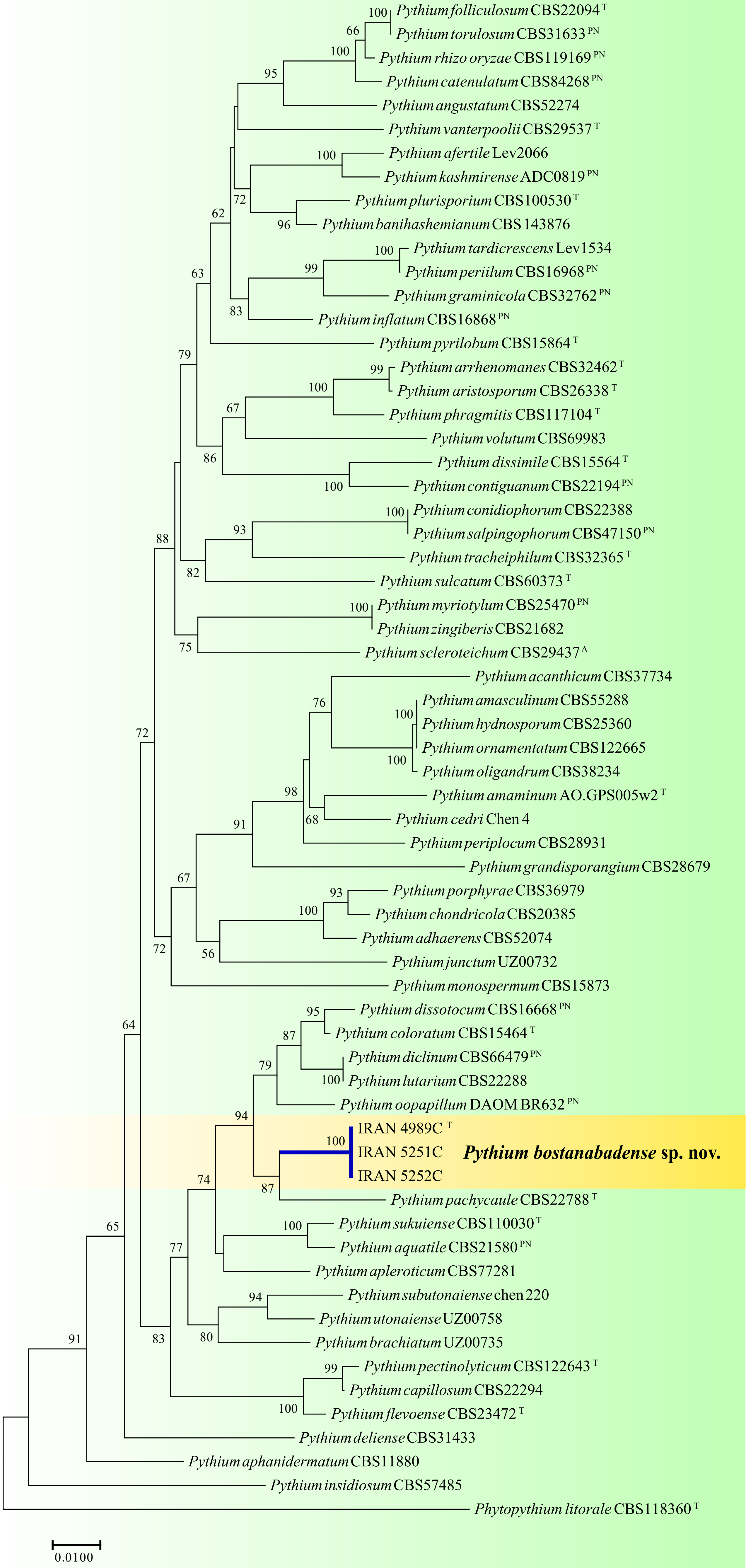

Figure 5. Minimum Evolution tree (ME) based on ***cox1*** sequence data for three examined strains and reference strains belonging to ***Pythium***. Numbers on the branches indicate bootstrap support in Minimum Evolution greater than 50%. *Phytopythium litorale* type strain CBS118360 is used as outgroup. T indicates ex-type strains; A authentic strain, identified by the author of the species and PN indicates authentic strains used for description in the monograph of van der Plaats-Niterink[21].

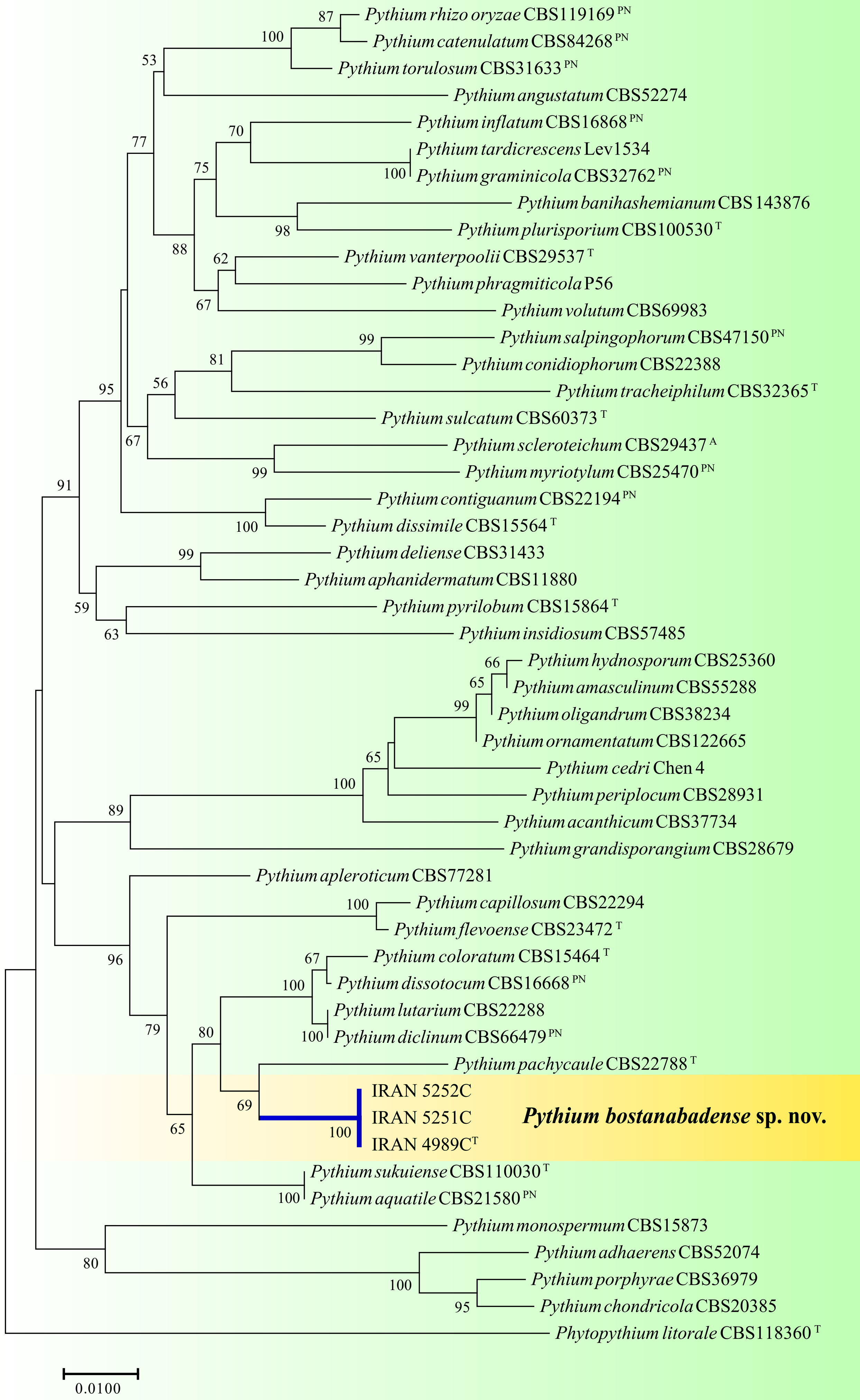

Figure 5. Minimum Evolution tree (ME) based on ***cox2*** sequence data for three examined strains and reference strains belonging to ***Pythium***. Numbers on the branches indicate bootstrap support in Minimum Evolution greater than 50%. *Phytopythium litorale* type strain CBS118360 is used as outgroup. T indicates ex-type strains; A authentic strain, identified by the author of the species and PN indicates authentic strains used for description in the monograph of van der Plaats-Niterink[21].
