# Supplementary figures and images for: Globisporangium tabrizense sp. nov., Globisporangium mahabadense sp. nov., and Pythium bostanabadense sp. nov. (Oomycota), three new species from Iranian aquatic environments
Source: Sci Rep. 2024 Dec 30;14:31701. doi: 10.1038/s41598-024-81651-0 (PMC11686014; doi:10.1038/s41598-024-81651-0)

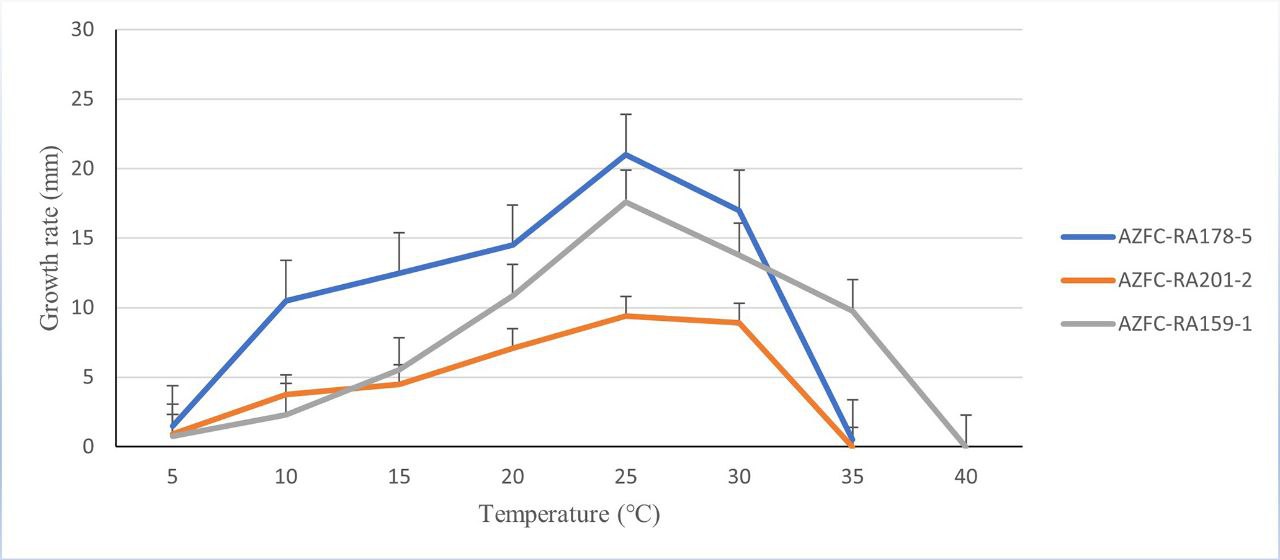

Supplement: Supplementary file 2 — Supplementary Material 2 [file 41598_2024_81651_MOESM2_ESM.jpg]
