# Supplementary material for: Globisporangium tabrizense sp. nov., Globisporangium mahabadense sp. nov., and Pythium bostanabadense sp. nov. (Oomycota), three new species from Iranian aquatic environments
Source: Sci Rep. 2024 Dec 30;14:31701. doi: 10.1038/s41598-024-81651-0 (PMC11686014; doi:10.1038/s41598-024-81651-0)
Supplement: Supplementary file 3 — Supplementary Material 3 [file 41598_2024_81651_MOESM3_ESM.docx]

**Supplementary Table S1.** A list of strains studied, with collection details and GenBank accession numbers.

| Species name | Sample code | locality | GenBank accession no. | | |
| --- | --- | --- | --- | --- | --- |
|  |  |  | ITS | *Cox1* | *Cox2* |
| *G. abappressorium*^T^ | CBS110198 | USA | HQ643408 | HQ708455 | KJ595409 |
| *G. acanthophoron* | CBS33729 | USA | HQ643413 | HQ708460 | KJ595376 |
| *G. acrogynum* ^A/T^ | CBS54988 | China | HQ643414 | HQ708461 | AB362324 |
| *G. alternatum* ^T^ | CBS139279 | - | AB998876 | AB998877 | - |
| *G. apiculatum* ^PN^ | CBS120945 | France | HQ643443 | HQ708490 | KJ595422 |
| *G. attrantheridium* | DAOM230383 | Canada | HQ643477 | HQ708524 | AB512886 |
| *G. baisense* ^T^ | QBS123 | - | FR775440 | FR774198 | - |
| *G. barbulae* ^T^ | CBS139569 | Japan | LC028389 | LC028392 | LC028395 |
| *G. breve* | HMAS 242231 | - | FR751317 | FR774196 | - |
| *G. buismaniae* | CBS28831 | Netherlands | HQ643479 | HQ708526 | KJ595368 |
| *G. camurandrum*^PN^ | DAOM BR876 | - | GQ244426 | GQ244425 | **-** |
| *G. canariense* ^T^ | CBS112353 | Spain | HQ643482 | HQ708528 | JX397983 |
| *G. capense* | CBS 149752 | Australia | OL342598 | OL331986 | OL332028 |
| *G. carolinianum* | CBS122659 | India | HQ643484 | HQ708530 | KJ595427 |
| *G. cederbergense* ^T^ | CBS133716 | South Africa | JQ412768 | JQ412793 | JQ412805 |
| *G. commune* | CBS 149753 | Australia | OL952618 | OL860920 | OL860929 |
| *G. coniferarum* ^T^ | CBS148568 | Iran | ON554847 | MZ020754 | MZ020759 |
| *G. cryptoirregulare* | CBS118731 | USA | HQ643515 | HQ708561 | GU071763 |
| *G. cylindrosporum* ^T^ | CBS21894 | Germany | HQ643516 | HQ708562 | GU071762 |
| *G. cystogenes* | CBS67585 | Netherlands | AY707985 | HQ708564 | KJ595396 |
| *G. debaryanum* | CBS75296 | United Kingdom | HQ643519 | HQ708565 | KJ595399 |
| *G. echinulatum*^PN^ | CBS28164 | Australia | HQ643531 | HQ708577 | AB362327 |
| *G. emineosum* | DAOM BR836 | - | GQ244428 | GQ244424 | - |
| *G. erinaceum* ^PN^ | CBS50580 | New Zealand | HQ643534 | HQ708578 | AB362326 |
| *G. glomeratum* | CBS122644 | France | HQ643542 | HQ708586 | KJ595424 |
| *G. heterothallicum*^T^ | CBS45067 | Canada | HQ643553 | HQ708597 | AB512919 |
| *G. hypogynum* | CBS23494 | France | HQ643565 | HQ708609 | AB362325 |
| *G. intermedium* | CBS26638 | Netherlands | HQ643572 | HQ708616 | AB507410 |
| *G. iranense*^T^ | IRAN2386C | Iran | MG182709 | MG182705 | - |
| *G. irregulare* | CBS250.28 | - | GQ410356 | GU071822 | GU071760 |
| *G. iwayamai* | CBS15664 | Australia | HQ643669 | HQ708713 | JX397979 |
| *G. izadpanahii* ^T^ | CBS144006 | Iran | MK454537 | OP321103 | MK455859 |
| *G. jasmonium* | DAOM229150 | USA | HQ643670 | HQ708714 | - |
| *G. kandovanense* | CBS 139567 | Iran | KP723167 | KP938427 | KP723171 |
| *G. kunmingense* ^T^ | CBS55088 | China | HQ643672 | HQ708716 | KJ595389 |
| *G. lacustre* | MAFF 236903 | Japan | LC209786 | LC209787 | - |
| *G. longandrum* ^PN^ | CBS112355 | France | HQ643679 | HQ708723 | KJ595413 |
| *G. longisporangium* ^PN^ | CBS122646 | France | HQ643680 | HQ708724 | KJ595426 |
| *G. lucens* | CBS113342 | Canada | HQ643681 | HQ708725 | KJ595415 |
| *G. macrosporum* | CBS57480 | Netherlands | HQ643684 | HQ708728 | AB512916 |
| *G. mahabadense* ^T^ | IRAN 4986C = AZFC-RAG201-2-1 | Iran | **PQ037626** | **PQ031213** | **PQ031206** |
|  | IRAN 5253C = AZFC-RAG201-2-2 | Iran | **PQ037627** | **PQ031212** | **PQ031205** |
| *G. mamillatum* | CBS25128 | Netherlands | HQ643687 | HQ708731 | AB512918 |
| *G. marsipium* | CBS77381 | Netherlands | HQ643690 | HQ708734 | KJ595401 |
| *G. mastophorum* | CBS37572 | United Kingdom | HQ643691 | HQ708735 | KJ595378 |
| *G. megalacanthum* | DAOM229154 | Germany | HQ643693 | HQ708737 | KJ595435 |
| *G. middletonii*^PN^ | CBS52874 | Netherlands | HQ643694 | HQ708738 | AB362318 |
| *G. minor*^T^ | CBS22688 | United Kingdom | HQ643696 | HQ708740 | AB362320 |
| *G. multisporum*^T^ | CBS47050 | - | HQ643700 | HQ708744 | AB362319 |
| *G. nagaii* | CBS77996 | United Kingdom | HQ643705 | HQ708749 | KJ595402 |
| *G. nodosum* ^T^ | CBS102274 | France | HQ643709 | HQ708753 | KJ595407 |
| *G. nunn* ^T^ | CBS80896 | USA | HQ643711 | HQ708755 | - |
| *G. okanoganense* ^T^ | CBS31581 | USA | HQ643714 | HQ708758 | KJ595373 |
| *G. ornacarpum* ^T^ | CBS112350 | France | HQ643721 | HQ708762 | KJ595411 |
| *G. orthogonon* | CBS37672 | Lebanon | HQ643723 | HQ708764 | KJ595379 |
| *G. paddicum* | CBS69883 | Japan | HQ643728 | JX397975 | JX397982 |
| *G. papilogynum* | CBS122648 | India | HQ643729 | HQ708770 | - |
| *G. paroecandrum* ^PN^ | CBS15764 | Australia | HQ643731 | HQ708772 | DQ071391 |
| *G. parvum*^T^ | CBS22588 | United Kingdom | HQ643738 | HQ708779 | AB362322 |
| *G. pengfuense* | Chen 93 | China | MF984129 | MF984166 | - |
| *G. perplexum* | CBS67485 | - | AY598658 | HQ708785 | KJ595395 |
| *G. pleroticum* | CBS77681 | Netherlands | HQ643748 | HQ708789 | AB362321 |
| *G. polare* ^T^ | CBS118203 | Norway | KJ716859 | - | KJ595417 |
| *G. polymastum* | CBS81170 | Netherlands | HQ643752 | HQ708793 | KJ595403 |
| *G. radiosum* | CBS21794 | France | HQ643756 | HQ708797 | KJ595356 |
| *G. recalcitrans* ^T^ | CBS 122440 | Spain | DQ357833 | EF426549 | KJ595423 |
| *G. rhizosaccharum*^T^ | CBS112356 | India | HQ643760 | HQ708801 | AB362323 |
| *G. rooibos* | STE-U7549 | - | JQ412770 | JQ412795 | JQ412807 |
| *G. rostratifingens*^PN^ | CBS115464 | USA | HQ643761 | HQ708802 | KJ595416 |
| *G. rostratum*^PN^ | CBS53374 | Netherlands | HQ643767 | HQ708808 | KJ595388 |
| *G. schmitthenneri* | Darke1611 | USA | JF836869 | JF895534 | JF895530 |
| *G. segnitium*^PN^ | CBS112354 | Spain | HQ643772 | HQ708813 | KJ595412 |
| *G. selbyi* | CBS129729 | USA | JF836871 | JF895536 | JF895532 |
| *G. solare* ^T^ | CBS119359 | Spain | KJ716860 | - | KJ595421 |
| *G. spiculum*^T^ | CBS122645 | France | HQ643790 | HQ708831 | KJ595425 |
| *G. spinosum* | CBS27667 | Netherlands | HQ643792 | HQ708833 | KJ595366 |
| *G. splendens* | CBS46248 | USA | HQ643795 | HQ708836 | AB512921 |
| *G. sylvaticum* ^T^ | CBS45367 | USA | HQ643845 | HQ708886 | - |
| *G. tabrizense* ^T^ | IRAN 4985C = AZFC-RAG178-5-1 | Iran | **PQ037624** | **PQ031210** | **PQ031204** |
|  | IRAN 5254C = AZFC-RAG175-5-2 | Iran | **PQ037625** | **PQ031211** | **PQ031203** |
| *G. takayamanum*^T^ | CBS122491 | Japan | HQ643854 | HQ708895 | - |
| *G. tenuihyphum* ^T^ | Chen 268 | China | MF984123 | MF984160 | - |
| *G. terrestris* | UM2097 | Australia | OL342605 | OL331993 | OL332034 |
| *G. ultimum* var. *sporangiiferum* ^T^ | CBS21965 | USA | HQ643879 | HQ708920 | AF196641 |
| *G. uncinulatum* | CBS51877 | Netherlands | HQ643944 | HQ708985 | KJ595385 |
| *G. urmianum* | IRAN2376C | Iran | KT894049 | KT894057 | - |
| *G. viniferum* | CBS119168 | Canada | HQ643956 | HQ708997 | KJ595419 |
| *G. violae* | CBS15964 | Australia | HQ643958 | HQ708999 | JX397980 |
| *G. yorkense* ^T^ | C12-118 | USA | KY990050 | KT692789 | KY985298 |
| *P. acanthicum* | CBS37734 | Sweden | AY598617 | HQ708456 | KJ595380 |
| *P. adhaerens* | CBS52074 | Netherlands | HQ643415 | HQ708462 | KJ595386 |
| *P. afertile* | Lev2066 | Canada | HQ643416 | HQ708463 | KJ595440 |
| *P. amaminum* ^T^ | AO.GPS005w2 | Japan | LC617877 | LC617875 | - |
| *P. amasculinum* | CBS55288 | China | HQ643434 | HQ708481 | KJ595390 |
| *P. angustatum* | CBS52274 | The Netherlands | HQ643437 | HQ708484 | KJ595387 |
| *P. aphanidermatum* | CBS11880 | France | HQ643438 | HQ708485 | KJ595344 |
| *P. apleroticum* | CBS77281 | The Netherlands | HQ643444 | HQ708491 | KJ595400 |
| *P. aquatile* ^PN^ | CBS21580 | United Kingdom | HQ643445 | HQ708492 | KJ595355 |
| *P. aristosporum*^T^ | CBS26338 | Canada | HQ643447 | HQ708494 | - |
| *P. banihashemianum* | CBS 143876 | Iran | KX228083 | OP321097 | KX228120 |
| *P. bostanabadense* ^T^ | IRAN 4989C = AZFC-RAP159-1 | Iran | **PQ037628** | **PQ031214** | **PQ031207** |
|  | IRAN 5251C = AZFC-RAP159-2 | Iran | **PQ037629** | **PQ031215** | **PQ031209** |
|  | IRAN 5252C = AZFC-RAP159-3 | Iran | **PQ037630** | **PQ031216** | **PQ031208** |
| *P. brachiatum* | UZ00735 | Japan | KJ995582 | KJ995592 | - |
| *P. capillosum* | CBS22294 | France | HQ643483 | HQ708529 | KJ595360 |
| *P. catenulatum*^PN^ | CBS84268 | USA | HQ643494 | HQ708540 | KJ595404 |
| *P. cedri* | Chen 4 | China | KX423748 | KX423742 | PP489327 |
| *P. chondricola* | CBS20385 | Netherlands | HQ643498 | HQ708544 | KJ595354 |
| *P. coloratum* ^T^ | CBS15464 | Australia | HQ643501 | HQ708547 | KJ595346 |
| *P. conidiophorum* | CBS22388 | United Kingdom | HQ643509 | HQ708555 | KJ595361 |
| *P. contiguanum*^PN^ | CBS22194 | Algeria | HQ643514 | HQ708560 | KJ595358 |
| *P. deliense* | CBS31433 | - | AY598674 | HQ708568 | KJ595372 |
| *P. diclinum*^PN^ | CBS66479 | The Netherlands | HQ643524 | HQ708570 | KJ595394 |
| *P. dissimile*^T^ | CBS15564 | Australia | HQ643526 | HQ708572 | KJ595347 |
| *P. dissotocum* ^PN^ | CBS16668 | USA | HQ643528 | HQ708574 | - |
| *P. flevoense*^T^ | CBS23472 | The Netherlands | HQ643538 | HQ708582 | KJ595363 |
| *P. folliculosum* ^T^ | CBS22094 | Switzerland | HQ643540 | HQ708584 | - |
| *P. graminicola*^PN^ | CBS32762 | Jamaica | HQ643545 | HQ708589 | AF196593 |
| *P. grandisporangium* | CBS28679 | USA | AY598692 | HQ708590 | KJ595367 |
| *P. hydnosporum* | CBS25360 | Germany | AY598672 | HQ708608 | KJ595364 |
| *P. inflatum*^PN^ | CBS16868 | USA | HQ643566 | HQ708610 | KJ595352 |
| *P. insidiosum* | CBS57485 | Costa Rica | HQ643570 | HQ708614 | KJ595391 |
| *P. junctum* | UZ00732 | Japan | KJ995576 | KJ995595 | - |
| *P. kashmirense*^PN^ | ADC0819 | India | HQ643671 | HQ708715 | - |
| *P. lutarium* ^T^ | CBS22288 | United Kingdom | HQ643682 | HQ708726 | KJ595359 |
| *P. monospermum* | CBS15873 | United Kingdom | HQ643697 | HQ708741 | KJ595350 |
| *P. myriotylum*^PN^ | CBS25470 | - | HQ643701 | HQ708745 | - |
| *P. oligandrum* | CBS38234 | United Kingdom | AY598618 | HQ708759 | KJ595381 |
| *P. oopapillum*^PN^ | DAOM BR632 | - | FJ655174 | FJ655178 | - |
| *P. ornamentatum* | CBS122665 | Turkey | HQ643722 | HQ708763 | KJ595428 |
| *P. pachycaule* ^T^ | CBS22788 | United Kingdom | HQ643724 | HQ708765 | KJ595362 |
| *P. pectinolyticum*^T^ | CBS122643 | France | HQ643739 | HQ708780 | - |
| *P. periilum*^PN^ | CBS16968 | USA | HQ643740 | HQ708781 | - |
| *P. periplocum* | CBS28931 | USA | AY598670 | HQ708784 | KJ595369 |
| *P. phragmiticola* | P56 | Germany | KC145165 | - | KC145166 |
| *P. phragmitis*^T^ | CBS117104 | Germany | HQ643746 | HQ708787 | - |
| *P. plurisporium*^T^ | CBS 100530 | USA | HQ643749 | HQ708790 | KJ595405 |
| *P. porphyrae* | CBS36979 | Japan | HQ643753 | HQ708794 | KJ595377 |
| *P. pyrilobum*^T^ | CBS15864 | Australia | HQ643755 | HQ708796 | KJ595349 |
| *P. rhizo-oryzae*^PN^ | CBS119169 | India | HQ643757 | HQ708798 | KJ595420 |
| *P. salpingophorum*^PN^ | CBS47150 | Germany | HQ643768 | HQ708809 | KJ595384 |
| *P. scleroteichum*^A^ | CBS29437 | USA | HQ643771 | HQ708812 | KJ595370 |
| *P. subutonaiense* | Chen 220a | China | MG654703 | MG674169 | - |
| *P. sukuiense*^T^ | CBS 110030 | Taiwan | HQ643836 | HQ708877 | KJ595408 |
| *P. sulcatum*^T^ | CBS60373 | USA | HQ643837 | HQ708878 | KJ595393 |
| *P. tardicrescens* | Lev1534 | USA | HQ643855 | HQ708896 | KJ595439 |
| *P. torulosum* ^PN^ | CBS31633a | The Netherlands | HQ643859 | HQ708900 | KJ595374 |
| *P. tracheiphilum*^T^ | CBS32365 | Italy | HQ643862 | HQ708903 | - |
| *P. utonaiense* | UZ00758 | - | KJ995586 | KJ995588 | - |
| *P. vanterpoolii* ^T^ | CBS29537 | United Kingdom | HQ643952 | HQ708993 | KJ595371 |
| *P. volutum* | CBS69983 | Japan | HQ643971 | HQ709012 | KJ595397 |
| *P. zingiberis* | CBS21682 | Japan | HQ643973 | HQ709014 | - |
| *Phytopythium littorale* ^T, O^ | CBS118360 | Germany | HQ643386 | HQ708433 | KJ595418 |

T and A indicate ex-type and authentic strains (respectively), identified by the author of the species and PN indicates authentic strains used for description in the monograph of van der Plaats-Niterink^21^.
