# Supplementary material for: Globisporangium tabrizense sp. nov., Globisporangium mahabadense sp. nov., and Pythium bostanabadense sp. nov. (Oomycota), three new species from Iranian aquatic environments
Source: Sci Rep. 2024 Dec 30;14:31701. doi: 10.1038/s41598-024-81651-0 (PMC11686014; doi:10.1038/s41598-024-81651-0)
Supplement: Supplementary file 4 — Supplementary Material 4 [file 41598_2024_81651_MOESM4_ESM.docx]

**Supplementary Table S2.** Detailed information of isolates obtained in this study.

| Isolate | Matrix | Ecosystem | Location | Longitude | Latitude | Date of collection |
| --- | --- | --- | --- | --- | --- | --- |
| IRAN 4989C^T^ = AZFC-RAP159-1 | Algae | Agricultural pool | Iran, East Azarbaijan, Bostan Abad, Qareh Baba | 37.775356 | 46.8767001 | Oct, 2022 |
| IRAN 5251C = AZFC-RAP159-2 | Algae | Agricultural pool | Iran, East Azarbaijan, Bostan Abad, Qareh Baba | 37.775356 | 46.8767001 | Oct, 2022 |
| IRAN 5252C = AZFC-RAP159-3 | Algae | Agricultural pool | Iran, East Azarbaijan, Bostan Abad, Qareh Baba | 37.775356 | 46.8767001 | Oct, 2022 |
| IRAN 4986C^T^ = AZFC-RAG201-2-1 | Algae | Irrigation water | Iran, West A., Mahabad | 36.905431 | 45.808550 | Oct, 2022 |
| IRAN 5253C = AZFC-RAG201-2-2 | Algae | Irrigation water | Iran, West Azarbaijan, Mahabad | 36.905431 | 45.808550 | Oct, 2022 |
| IRAN 4985C^T^ = AZFC-RAG178-5-1 | Root of *Cynodon dactylon* | Irrigation water | Iran, East Azarbaijan. Tabriz, Varanaq | 37.9985651 | 46.1771981 | Oct, 2022 |
| IRAN 5254C = AZFC-RAG178-5-2 | Root of *Cynodon dactylon* | Irrigation water | Iran, East Azarbaijan. Tabriz, Varanaq | 37.9985651 | 46.1771981 | Oct, 2022 |
