# Supplementary material for: Globisporangium tabrizense sp. nov., Globisporangium mahabadense sp. nov., and Pythium bostanabadense sp. nov. (Oomycota), three new species from Iranian aquatic environments
Source: Sci Rep. 2024 Dec 30;14:31701. doi: 10.1038/s41598-024-81651-0 (PMC11686014; doi:10.1038/s41598-024-81651-0)
Supplement: Supplementary file 5 — Supplementary Material 5 [file 41598_2024_81651_MOESM5_ESM.docx]

**Supplementary Table S3a.** Morphological comparison of *Globisporangium tabrizense* sp. nov. and related species.

| Characteristics | *G. tabrizense* sp. nov. | *G. lucens* (Ali-Shtayeh and Dick 1985, Uzuhashi et al. 2010) | *G. viniferum* (Paul and Bala 2008) | *G. debaryanum*  (Hendrix Jr 1974) |
| --- | --- | --- | --- | --- |
| Daily growth on PCA | 21 mm | 9 mm | 25 mm | 30 mm |
| Width of main hyphae | 2.5-8.5 µm in diameter | 3.5–6.5 µm in diameter | > 7 µm in diameter | - |
| Sporangium | Terminal and intercalary, lemon-shaped, filamentous, globose, 8.5-39.5 µm in diameter | Globose or subglobose, terminal and occasionally catenulate 2–3 in a chain, intercalary (21–25 µm in diameter); discharge tube up to 30 µm in diameter long, 1–2 per sporangium; Encysted zoospores 8–10 µm in diameter | Terminal and intercalary, spherical (7–25 μm in diameter), ovoid to elongated; at different temperatures, in sterile distilled, pond, and soil extract water | Spherical and terminal, up to 21 µm in diameter |
| Zoospores | Not observed | 8-10 µm in diameter | Zoospores were never observed in spite of repeated culturing | Present |
| Oogonia | Intercalary and terminal, globose, smooth, 13.5-22 µm in diameter, rarely 2 in chain | Smooth walled, globose, 22–35 µm in diameter, rarely pyriform, usually terminal, occasionally intercalary, rarely 2–3 in chain | Mostly intercalary, occasionally terminal, sometimes catenulate, 17–29 μm in diameter, antheridia and oogonia borne on an appressorium, cylindrical or peanut-shaped | Spherical, smooth, 13-22 µm in diameter, terminal |
| Antheridia | Diclinous and monoclinous, 1-2 per oogonium | 1–2(5) per oogonium, monoclinous, usually stalked, originating usually more than 20 µm distance from the oogonium base, occasionally diclinous, antheridial cells clavate, occasionally 2–3 borne on one antheridial branch | Hypogynous, monoclinous sessile or monoclinous on short branches, at times diclinous, 1–5 per oogonium, antheridial cells conspicuous and at times bi-lobed, monoclinous stalked antheridia making a broad apical contact zone with the oogonia | 1 per oogonium, arising from the oogonial stalk |
| Oospore | Single, rarely 2, globose, plerotic or aplerotic, 12.5-22 µm in diameter | Aplerotic, usually single, occasionally 2 oospores per oogonium, globose, 17–23 µm in diameter. | Spherical, elongated and irregular, 1 to 3 per oogonia, plerotic, rarely aplerotic, 12-22 µm in diameter | Plerotic rarely aplerotic, Mostly spherical, single, 12-21 µm in diameter |
| Wall thickness of oospore | 1.2-1.8 µm | 1.5-2.5 µm | 1-2 µm | 1 µm |

**Supplementary Table S3b.** Morphological comparison of *Globisporangium mahabadense* sp. nov. and related species.

| **Characteristics** | ***G. mahabadense* sp. nov.** | ***G. urmianum* (Abrinbana et al. 2016)** | ***G. longisporangium* (Paul et al. 2005)** | ***G. longandrum* (Paul 2001)** |
| --- | --- | --- | --- | --- |
| **Daily growth on PCA** | 9.5 mm |  | 11 mm | 7 mm |
| **Width of main hyphae** | 2.5-5 µm in diameter | 7 µm in diameter | 6-8 µm in diameter | 7-8 µm in diameter |
| **Sporangium** | Globose, terminal or intercalary, 11-33 µm in diameter | Mostly globose at times subglobose, ellipsoidal, elongated, ovoid or pyriform, mostly terminal, occasionally intercalary, sometimes in chains of 2 | Terminal or intercalary, globose, cylindrical, peanut, and oval | Globose to elongated, mostly intercalary and catenulate, but at times terminal and subterminal, 16-36 µm in diameter |
| **Zoospores** | Non-observata | Non-observata | Non-observata | Non-observata |
| **Oogonia** | Oogonium is rarely formed, terminal and intercalary, smooth, globose, 14-23 µm in diameter | Globose, rarely elongated, smooth-walled or rarely with a projection, terminal, occasionally subterminal or intercalary, terminal oogonia sometimes on short side branches of hyphae, rarely lateral or on intercalary and hypogynous antheridia | Smooth, globose, terminal and intercalary, 13–23 µm in diameter | Smooth, spherical, at times elongated, terminal, subterminal or intercalary, 18-26 µm in diameter, coarsely granulated protoplasm. |
| **Antheridia** | Diclinous, one per oogonium | 1-2 per oogonium, mostly hypogynous, occasionally intercalary and hypogynous | Hypogynous or monoclinous sessile, 1-3 per oogonium, diclinous, antheridial cells at times catenulate | Hypogynous, monoclinous, very long and longitudinally, inflated, bilobed into two conspicuous cells |
| **Oospore** | Globose and semi-globose, plerotic, 16-23 µm in diameter | Globose, occasionally subglobose, rarely peanut-shaped, plerotic occasionally aplerotic, usually one but rarely two per oogonium | Usually one, frequently 2 and rarely 3 per oogonium, plerotic, 12–22 µm in diameter | Spherical, plerotic or aplerotic, usually one but rarely two oospores per oogonium 18-23 µm in diameter |
| **Wall thickness of oospore** | 1-3 µm | up to 2 µm | 1-15 µm | 1-2.5 µm |

**Supplementary Table S3c.** Morphological comparison of *Pythium* *bostanabadense* sp. nov. and related species.

| Characteristics | *Pythium* *bostanabadense* sp. nov. | *Pythium pachycaule* (Ali-Shtayeh and Dick 1985) |
| --- | --- | --- |
| Daily growth on PCA | 17.5 | 15.4 |
| Minimum temperature for mycelial growth | 2 ℃ | 5 ℃ |
| Optimum temperature for mycelial growth | 25 ℃ | 25 ℃ |
| Maximum temperature for mycelial growth | 35 ℃ | 37 ℃ |
| Width of main hyphae | 1.4-4 µm in diameter | 4–10 µm in diameter |
| Sporangium | Globose, intercalary and terminal | Filamentous, terminal, slightly swollen |
| Zoospores | 6-12 µm in diameter, at temperature of 20-25 ℃ | 7-12 µm in diameter |
| Oogonia | Intercalary and terminal, smooth, globose, 17.5–24 µm in diameter | Smooth, globose or trumpet shaped, with a long thick neck cell, terminal and intercalary, 24-34 µm in diameter |
| Antheridia | 1 and rarely 2 per oogonium, diclinous | Diclinous and monoclinous, 1-3 per oogonium |
| Oospore | Globose, aplerotic, 14.5-20 µm in diameter | Single, rarely two, globose, occasionally spindle-shaped, 1-2 per oogonium, 18-25 µmin diameter, aplerotic |
| Wall thickness of oospore | 1.5-3.5 µm | 1.5 – 3 µm |
