# Supplementary material for: Globisporangium tabrizense sp. nov., Globisporangium mahabadense sp. nov., and Pythium bostanabadense sp. nov. (Oomycota), three new species from Iranian aquatic environments
Source: Sci Rep. 2024 Dec 30;14:31701. doi: 10.1038/s41598-024-81651-0 (PMC11686014; doi:10.1038/s41598-024-81651-0)
Supplement: Supplementary file 6 — Supplementary Material 6 [file 41598_2024_81651_MOESM6_ESM.docx]

**Supplementary Table S4.** Pairwise comparison of the loci analyzed for novel species described in the current study with closely related taxa.

| **Novel species** | **Closely related taxa** | **Loci, identity and percentage and gaps** | | | | | | | |
| --- | --- | --- | --- | --- | --- | --- | --- | --- | --- |
|  |  | **ITS** | |  | ***COX1*** | |  | ***COX2*** | |
|  |  | **Identity and**  **percentage** | **Gaps** |  | **Identity and**  **percentage** | **Gaps** |  | **Identity and**  **percentage** | **Gaps** |
|  |  |  |  |  |  |  |  |  |  |
| *Globisporangium tabrizense* sp. nov. (IRAN 4985C) | *G. lucens* CBS113342 | 777/817 (95%) | 21/817(0%) |  | 493/494 (99%) | 0/494(0%) |  | 417/419 (99%) | 0/419 (0%) |
|  | *G. viniferum* CBS119168 | 796/813 (97%) | 3/813 (0%) |  | 478/480 (99%) | 0/480(0%) |  | 413/419 (98%) | 0/419 (0%) |
|  | *G. debaryanum* CBS75296 | 807/812 (99%) | 2/812 (0%) |  | 478/480 (99%) | 0/480(0%) |  | 413/419 (98%) | 0/419 (0%) |
|  |  |  |  |  |  |  |  |  |  |
|  |  |  |  |  |  |  |  |  |  |
| *G. mahabadense* sp. nov. (IRAN 4986C) | *G. longisporangium* CBS122646 | 797/800 (99%) | 0/800 (0%) |  | 486/494 (98%) | 0/494 (0%) |  | 523/526 (99%) | 0/526 (0%) |
|  | *G. urmianum* IRAN2376C | 787/800 (98%) | 1/800 (0%) |  | 480/494 (97%) | 0/494 (0%) |  | ‒ | ‒ |
|  | *G. longandrum* CBS112355 | 787/800 (98%) | 1/800 (0%) |  | 479/494 (96%) | 0/494 (0%) |  | 415/419 (98%) | 1/526 (0%) |
|  | *G. selbyi* CBS129729 | 777/800 (97%) | 4/800 (0%) |  | 481/494 (97%) | 0/494 (0%) |  | 512/530 (96%) | 1/530 (0%) |
|  |  |  |  |  |  |  |  |  |  |
|  |  |  |  |  |  |  |  |  |  |
| *Pythium bostanabadense* sp. nov. (IRAN 4989C) | *Pythium pachycaule* CBS22788 | 759/781 (97%) | 5/781 (0%) |  | 530/550 (96%) | 0/550 (0%) |  | 485/504 (96%) | 0/504 (0%) |
|  |  |  |  |  |  |  |  |  |  |
